# Electrochemical and Mechanical Evolution of Sulfide‐Based Solid Electrolytes: Insights from Operando XPS and Cell Pressure Measurements

**DOI:** 10.1002/smll.202508796

**Published:** 2025-10-03

**Authors:** Valerie Siller, Linfeng Xu, Laurent Castro, Aurélie Guéguen, Mario El Kazzi

**Affiliations:** ^1^ PSI Center for Energy and Environmental Sciences Paul Scherrer Institut Forschungsstrasse 111 Villigen CH‐5232 Switzerland; ^2^ Toyota Motor Europe Hoge Wei 33 Zaventem B‐1930 Belgium

**Keywords:** all‐solid‐state battery, interphase electrical properties, Li_3_PS_4_ solid electrolyte, operando cell pressure monitoring, operando X‐ray photoelectron spectroscopy

## Abstract

Understanding the electrochemical and mechanical behavior of solid electrolytes beyond their electrochemical stability window is crucial for enabling high energy density all‐solid‐state batteries. Accordingly, this work systematically studies a model working electrode of Li_3_PS_4_, ball milled with vapor grown carbon fiber (VGCF). *Operando* X‐ray photoelectron spectroscopy can identify and quantify the potential‐dependent redox byproducts, their reversibility, and electrical properties, while *operando* cell pressure measurements correlate these with volume changes and mechanical instability. The study examines voltages up to 5.0 V and down to −0.05 V versus Li/Li^+^, mimicking cathode and anode cycling. It demonstrates that within the 2.4–5.0 V region, Li_3_PS_4_ oxidation byproducts are primarily polysulfides composed of bridging sulfurs (P‐S‐S‐P) between PS_4_^3‐^ units, free of elemental sulfur (S^0^), and electrically conductive. The Li_3_PS_4_ oxidation process occurs at 2.8 V during first charge and ends at 3.4 V, with volume shrinkage at the VGCF interface. During reduction (2.4 to −0.05 V), polysulfides convert reversibly to Li_3_PS_4_ between 1.9 and 1.7 V, then to Li_2_S and Li_n_P (0 ≤ n ≤ 3) between 1.9 and 0.6 V, causing volume expansion and the transition to an electrically insulating interphase. Below 0.6 V, Li_2_O formation dominates without further evolution of Li_2_S or Li_n_P.

## Graphical abstract

This research explores the electro‐chemo‐mechanical properties of Li_3_PS_4_ solid electrolytes in all‐solid‐state batteries beyond their electrochemical stability window. Employing *operando* X‐ray photoelectron spectroscopy and *operando* pressure measurements, it identifies and quantifies the Li_3_PS_4_ redox byproducts, their onset‐potentials and reversibility, alongside significant volume changes and the changes in the electrical properties of the interphase.

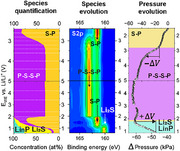

## 1 Introduction

Lithium‐ion batteries (LIBs), widely used as rechargeable high energy density storage for portable electronics, electromobility, biomedical implants, and stationary applications, are increasingly being considered for replacement with all‐solid‐state batteries (ASSBs).^[^ ^1^, ^2^, ^3^, ^4^ ^]^ By substituting the conventional flammable carbonate‐based liquid electrolytes with non‐flammable solid electrolytes (SEs), which possess a comparable ionic conductivity and higher transference number equal to unity, ASSBs can hold several promises like: (i) unlocking the common trade‐off relationship between high energy and high power densities,^[^ ^5^ ^]^ which allows fast charging rates and (ii) improves safety by overcoming the prevailing intrinsic fire and explosion risks.^[^ ^6^, ^7^ ^]^ Furthermore, the adequate inherent stiffness of inorganic SEs renders them compatible with metallic lithium. It is expected to effectively suppress the growth of lithium (Li) dendrites to a considerable extent, permitting them to achieve high energy density (≈500 Wh kg^−1^) when combined with a thin Li film as the anode,^[^ ^8^ ^]^ or the Li reservoir‐free anode.^[^ ^9^, ^10^ ^]^

Sulfide‐based SEs are among the most promising; they have gained great popularity as they offer high ionic conductivities beyond 10^−2^ S cm^−1^ at room temperature, together with an ease of synthesis and processability.^[^ ^2^, ^11^, ^12^ ^]^ Nevertheless, sulfide‐based ASSBs can suffer from some major limitations that need to be fundamentally understood and surmounted before their commercialization.^[^ ^13^, ^14^ ^]^ One of the key limitations is the interfacial electro‐chemo‐mechanical instability between the SEs and the active materials of the cathode and anode. This instability often leads to a significant increase in cell impedance, which hinders the lithium ion transport kinetics and ultimately deteriorates the overall electrochemical performance.^[^ ^15^ ^]^

In the last decade, tremendous efforts have been made by several research groups with the purpose of elucidating the (electro‐) chemical redox mechanisms of the sulfide‐based SEs, clarifying their thermodynamic stability, identifying the reversible oxidation and reduction byproduct species,^[^ ^16^, ^17^, ^18^ ^]^ accurately defining their redox onset potentials,^[^ ^19^, ^20^, ^21^ ^]^ and understanding their chemical reaction with the active materials.^[^ ^22^, ^23^, ^24^ ^]^ Extensive research has suggested a multitude of different reaction pathways for the SE decomposition, based on computational methods,^[^ ^25^, ^26^, ^27^ ^]^ electrochemical characterization,^[^ ^28^, ^29^ ^]^ and various ex situ and *post mortem* characterization techniques.^[^ ^16^, ^19^, ^27^, ^30^, ^31^, ^32^, ^33^, ^34^, ^35^ ^]^ Moreover, efforts have been made to visualize, quantify, and understand the mechanisms behind mechanical instabilities, such as cracks and void formation, and the subsequent contact loss between the SE and the cathode or anode active materials.^[^ ^36^, ^37^ ^]^

In general, sulfide‐based SEs exhibit a narrow electrochemical stability window. For instance, first‐principles calculations indicate that Li_3_PS_4_ (LPS) is electrochemically oxidized above 2.3–2.5 V versus Li/Li^+^ and reduced below 2.0–1.7 V versus Li/Li^+^.^[^ ^25^, ^26^, ^38^ ^]^ However, experimentally the onset redox potentials diverge, as there is no standardization in the materials and in the employed methods, but usually remain in the same ranges as the theoretical calculations.^[^ ^16^, ^18^, ^19^, ^28^, ^39^ ^]^ Furthermore, there is an inconsistent consensus regarding the redox‐reaction mechanisms to describe the redox byproducts. On the one hand, the first‐principles calculations propose two conversion reaction paths for Li_3_PS_4_ on oxidation and reduction. The first one is from *Zhu* et al.,^[^ ^40^ ^]^ where on oxidation the LPS is converted to P_2_S_5_ and S^0^ (Equation 1) while on reduction is converted to Li_2_S and Li_3_P (Equation 3). The second one is from *Richards* et al.,^[^ ^38^ ^]^ where on oxidation the LPS is converted to Li_4_P_2_S_6_ and S^0^ (Equation 2), while on reduction it is converted to Li_4_P_2_S_6_ and Li_2_S (Equation 4). On the other hand, experimentally, the redox reaction of LPS has been demonstrated to be partially reversible.^[^ ^18^, ^19^, ^28^, ^33^ ^]^ However, there remains some disagreement regarding the exact oxidation and reduction byproducts and their corresponding redox pathways. For example, *Hakari* et al.^[^ ^16^ ^]^ do not detect elemental sulfur (S^0^) during the oxidation of LPS, neither in the XAS sulfur K‐edge, nor in the XPS S2p core level, disagreeing with both Equations 1 and 2 and proposing a third mechanism described as the association and dissociation of the bridging P‐S−S‐P bonds between the PS_4_ units of the LPS occurring during the Li extraction−insertion processes. In contrast, other groups claim to detect elemental sulfur (S^0^) on oxidation by fitting the XPS S2p in three components associated with PS_4_^3‐^ (S‐P), P‐S_x_‐P (1 ≤ x ≤ 2), and S^0^ species. This suggests possible agreement with Equations 1 and 2, where S^0^ is formed in addition to P_2_S_5_, or two interconnected PS_4_ units with bridging sulfur (‐S‐S‐), which can also disproportionate to P_2_S_7_^4‐^ or P_2_S_6_^2‐^ polysulfides, depending on the level of Li‐extraction.^[^ ^17^, ^18^, ^19^, ^33^, ^41^ ^]^

*Oxidation*:

(1) $$2Li_{3}PS_{4}\to P_{2}S_{5}+3S^{0}+6Li^{+}+6e^{-}$$

(2) $$2Li_{3}PS_{4}\to Li_{4}P_{2}S_{6}+2S^{0}+2Li^{+}+2e^{-}$$

*Reduction*:

(3) $$2Li_{3}PS_{4}+16Li^{+}+16e^{-}\to 8Li_{2}S+2Li_{3}P$$

(4) $$2Li_{3}PS_{4}+2Li^{+}+2e^{-}\to Li_{4}P_{2}S_{6}+2Li_{2}S$$

Further discrepancy is also observed in the detected reduction byproducts of LPS. Several groups manage to detect both the Li_2_S and the Li_n_P (0 ≤ n ≤ 3) species in the XPS S2p and P2p core levels during LPS reduction,^[^ ^18^, ^19^, ^21^ ^]^ confirming partially what is expected in Equation 3 and Equation 4.^[^ ^38^, ^40^ ^]^ However, some reported papers could not detect the Li_n_P species,^[^ ^17^, ^42^ ^]^ but rather reversible conversion of PS_4_^3‐^ to P_2_S_6_^4‐^ based on the Raman spectroscopy, supporting more Equation 4.^[^ ^41^ ^]^

The detection of elemental S^0^ on oxidation and elemental P^0^ or Li_n_P on reduction can be challenging by XPS without remaining ambiguity, as there is either (i) a close local environment like in the case of sulfur between bridging sulfur and elemental S^0^, leading to an overlap or small shift in the S2p binding energies,^[^ ^17^, ^18^, ^19^, ^43^ ^]^ or (ii) as in the case of phosphorus, where the P2p core level signal intensity is often weak in the sulfur‐based SE, and multiple partly reduced phosphorus species Li_n_P of similar structure coexist in parallel with all their different phosphorus oxidation states.^[^ ^30^, ^44^, ^45^ ^]^

Despite these endeavors, significant challenges persist in understanding the redox mechanisms of sulfide‐based SEs. First, probing the buried electrified interface of a working electrode is inherently difficult, particularly under *operando* conditions. Second, the complexity of the electro‐chemo‐mechanical reactions, which occur at the nanoscale, complicates their detection. As a result, several key aspects remain elusive, including (i) the identification and quantification of redox byproducts and their potential‐dependent changes, (ii) the byproducts electrical and ionic properties evolution throughout cell operation, and (iii) the quantification of volume changes during redox byproduct formation, that can lead to the loss of particle contact within the working electrode.

To address the key aspects mentioned above, *operando* X‐ray photoelectron spectroscopy (XPS) is employed as the technique of choice, providing both non‐destructive and time‐resolved analysis of the redox reaction mechanism under realistic operation conditions.^[^ ^21^, ^42^, ^46^, ^47^, ^48^, ^49^ ^]^ It ensures access to the electrified electrode‐electrolyte interface and measures its changes under ultra‐high vacuum (UHV) during battery cycling, always at the same surface environment of the working electrode. For sulfide‐based SEs, *operando* XPS offers good intensity and enough binding energy resolution of the S2p, P2p, and Li1s core levels. This allows for accurate determination of their local chemical changes by monitoring the binding energy shifts without the risk of surface parasitic reactions or modification, as demonstrated for the *post mortem* analysis.^[^ ^46^ ^]^ In addition, the core level peak alignment and component assignment are more precise, as the sample‐to‐sample deviation and possibility of charging effects are mitigated. Finally, *operando* XPS enables a real‐time correlation between the chemical species and their electrical properties by measuring the local surface potential, sensitive to the core level binding energy shifts with the applied voltage.^[^ ^50^, ^51^, ^52^, ^53^, ^54^ ^]^

In this study, we conducted *operando* XPS on a model working electrode made from Li_3_PS_4_ (LPS), which is representative of the sulfide‐based SE family, and was mixed with vapor grown carbon fiber (VGCF) using a ball milling process. Both high voltage range up to 5.0 V versus Li/Li^+^ and low voltage range down to −0.05 V versus Li/Li^+^ have been investigated to mimic the cathode and anode cycling potential windows, respectively. The intention of this cycling protocol is to precisely determine the onset oxidation and reduction potentials and identify and quantify the redox byproducts and their dynamic mutual interplay and reversibility. By leveraging the customized *operando* XPS cell dedicated to ASSB studies, first introduced by *Wu* et al.,^[^ ^55^ ^]^ we could successfully monitor the dynamic (electro‐) chemical changes at the LPS/VGCF interface and create a direct link between the newly formed chemical species and their electrical properties, realized through the accurate voltage and current control over the cell cycling. This is particularly important at high voltages, where ambiguity remains regarding the formation of elemental sulfur, supposed to lead to an electrically insulating interface, and at the low voltages, where changes in the phosphorus environment are often poorly resolved in existing *post mortem* studies.^[^ ^18^, ^19^, ^29^ ^]^ The challenge also extends to in situ studies, which typically involve direct lithium deposition onto the sample surface rather than electrochemical plating.^[^ ^21^, ^45^, ^56^, ^57^, ^58^ ^]^ This has led to unclear species assignment and quantification at the low voltage interface, which fails to resolve the full mechanism of phosphorus reduction in the sulfide SE degradation mechanism. Further, the electrical properties of many degradation byproducts remain poorly understood due to the challenge of obtaining proper references. *Operando* XPS can identify these properties since the working electrode is grounded to the analyzer,^[^ ^55^ ^]^ allowing for the observation of electrical properties of the voltage‐dependent interface species.

Additionally, *operando* cell pressure measurements enable us to establish a direct correlation between the redox byproduct species and volume changes/mechanical instability of the working electrode. These changes are associated with the shrinkage and expansion of the oxidized and reduced LPS, respectively, at the interface with VGCF. The correlation between *operando* XPS species evolution and *operando* pressure monitoring for lithium thiophosphate SEs operating outside their thermodynamic electrochemical stability window has not been previously established.

## 2 Results and Discussion

### 2.1 LPS:VGCF WE Morphology and Cycling Performance

**Figure 1:**
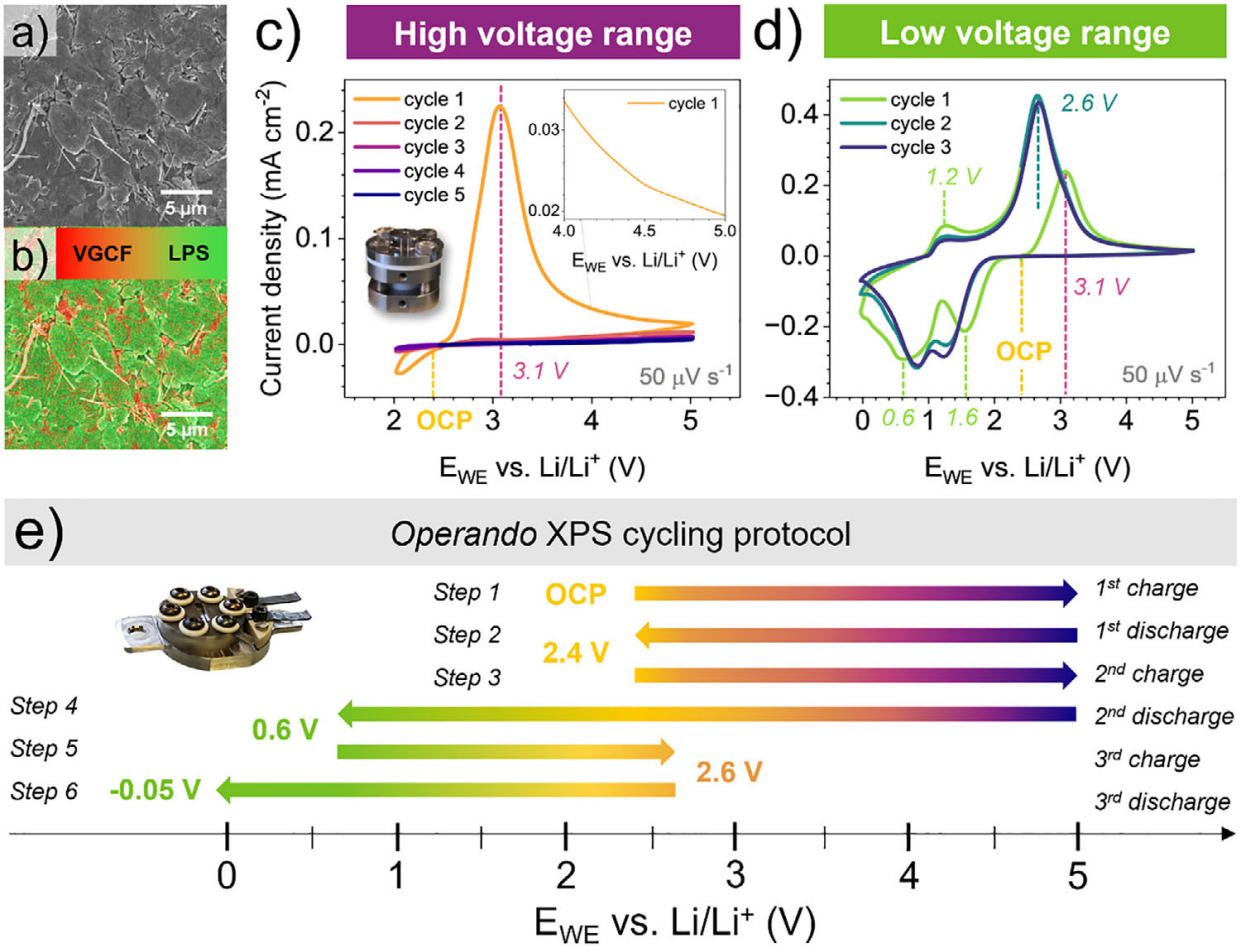
SEM In‐lens image (white scale bar 5 µm) of LPS:VGCF cathode composite pressed at 400 MPa in (a) and (b) showing the corresponding elemental map of carbon (VGCF) and sulfur (LPS) obtained by EDX. In (c‐d) cyclic voltammetry of the LPS:VGCF cathode inside a standard cell (see inset) is compared between c) the high and d) low voltage range. In e) the cycling protocol of the operando XPS cell (see inset) is shown for each charge/discharge cycle, introducing the color code assigned in the following analysis for each voltage range.

The SEM surface morphology (**Figure** **1**a) and EDX elemental mapping (Figure 1b) of the ball milled LPS:VGCF (80:20 wt %) WE show a dense surface and good dispersion and interconnected network between the LPS and the VGCF, securing good electrical percolation within the WE and helping to effectively oxidize and reduce a large amount of LPS electrochemically. To mimic the high‐voltage cathode and low‐voltage anode operating windows, CV measurements were conducted on the LPS:VGCF WE within two different voltage ranges to accurately identify the redox currents and reversibility. In the high voltage range, five cycles are performed starting at the OCP of 2.4 V followed by a positive sweep up to 5.0 V (Figure 1c), then reversed to a negative sweep to 2.0 V. During the first charge (positive sweep) one main oxidation peak is observed at 3.1 V assigned to the LPS oxidation. Thereafter, the oxidation current density drops rapidly below 0.1 mA cm^−2^, indicating the end of the oxidation reaction. The corresponding calculated specific first charge capacity is 86 mAh g^−1^ normalized on the LPS mass (Figure S1, Supporting Information). During the first discharge (negative sweep), the reductive current remains below 0.01 mA cm^−2^ without any detectable reduction peak until 2.4 V. Between 2.4 and 2.0 V, a small reductive current of a maximum 0.03 mA cm^−2^ is observed, suggesting the onset potential of the reversible redox process of the oxidized LPS byproducts. The subsequent charge and discharge cycles in the high voltage range reveal no significant progress on the LPS oxidation. Entering the low voltage range (see Figure 1d), three cycles are performed starting at OCP of 2.4 V with a positive sweep up to 5.0 V and then reversed with a negative sweep to 0.0 V. During the first discharge, two main reduction peaks are observed reaching a maximum reductive current at 1.6 and 0.6 V. The specific discharge capacity calculated at 0.0 V is 218 mAh g^−1^ normalized on the LPS mass. During the second charge cycle, two prominent oxidation peaks are observed: a minor peak at 1.2 V and a more pronounced peak at 2.6 V. These peaks are attributed to a reversible redox process of the reduced LPS byproducts. For the subsequent charge and discharge cycles the same redox peaks are perceived, confirming the overall reversibility of the redox reactions over long cycling.

### 2.2 Operando XPS

#### 2.2.1 Cycling Protocol

The electrochemical cycling reliability of the *operando* XPS cell compared to the standard electrochemical cell is evaluated inside the glovebox by performing CV measurements over the full voltage range of 0.6–5.0 V (see Figure S2, Supporting Information). The same redox peaks are observed on both cells, with slight overpotentials of 50 mV on oxidation and 64 mV on reduction for the *operando* cell. This mainly results from the lower cycling pressure applied to the *operando* XPS cell, in comparison to the standard electrochemical cells.

The cycling protocol employed for the *operando* XPS measurements is presented in Figure 1e, designed to mimic both the oxidation (high voltage range) and reduction (low voltage range) reaction mechanisms of LPS in six different cycling steps. To study the high voltage range oxidation mechanisms, step 1 starts with the first charge from OCP at 2.4 V up to 5.0 V followed by step 2, first discharge from 5.0 to 2.4 V, then step 3, a second charge from 2.4 to 5.0 V. Step 1 provides the oxidation mechanism of LPS, essentially the onset oxidation potential, the oxidation byproducts identification and their dynamic evolution up to 5.0 V. Step 2 offers insights into the redox reversibility during discharge and step 3 evaluates the possibility of further oxidation on the second charge. In the study of the low voltage reduction mechanisms, step 4 represents the second discharge from 5.0 V until 0.6 V, proceeded with step 5, as third charge from 0.6 up to 2.6 V and finishing with step 6, third discharge from 2.6 V until −0.05 V. Step 4 instructs us about the reversible redox process during the reduction of LPS and the oxidized LPS byproducts. We limited step 4 to 0.6 V, as an intermediate potential before risking any undesirable reactions arising from the remaining background gas pressure in the UHV, like the formation of Li_2_O/Li_2_CO_3_,^[^ ^49^, ^54^, ^59^, ^60^ ^]^ and lithium plating at much lower potentials. The UHV (low 10^−9^ mbar) still comprises residual gases, that is, traces of water vapor, CO/CO_2_, nitrogen and oxygen. As we know that Li_2_O forms immediately when plating metallic Li, its reaction to form Li_2_CO_3_ is very likely, as Li_2_O is prone to quickly react to carbonates in the presence of adhering (surface adventitious carbon)^[^ ^61^ ^]^ or surrounding CO or CO_2_.^[^ ^49^ ^]^ However, step 5 is expected to reveal the reversibility of the reduced byproducts, and step 6 explores the impact of the extremely low potentials and possible Li plating below 0.0 V.

#### 2.2.2 Operando XPS in a High Voltage Range of up to 5.0 V

The *operando* XPS is performed on the LPS:VGCF WE, while cycling in potentiostatic steps and simultaneous core level spectra acquisition every 200 or 400 mV in step 1, 200 or 500 mV in step 2, and 500 or 600 mV in step 3. Figure S3, Supporting Information presents the selected potential steps, the current response, and the specific charge retrieved during the *operando* XPS measurements in steps 1, 2, and 3.

**Figure 2:**
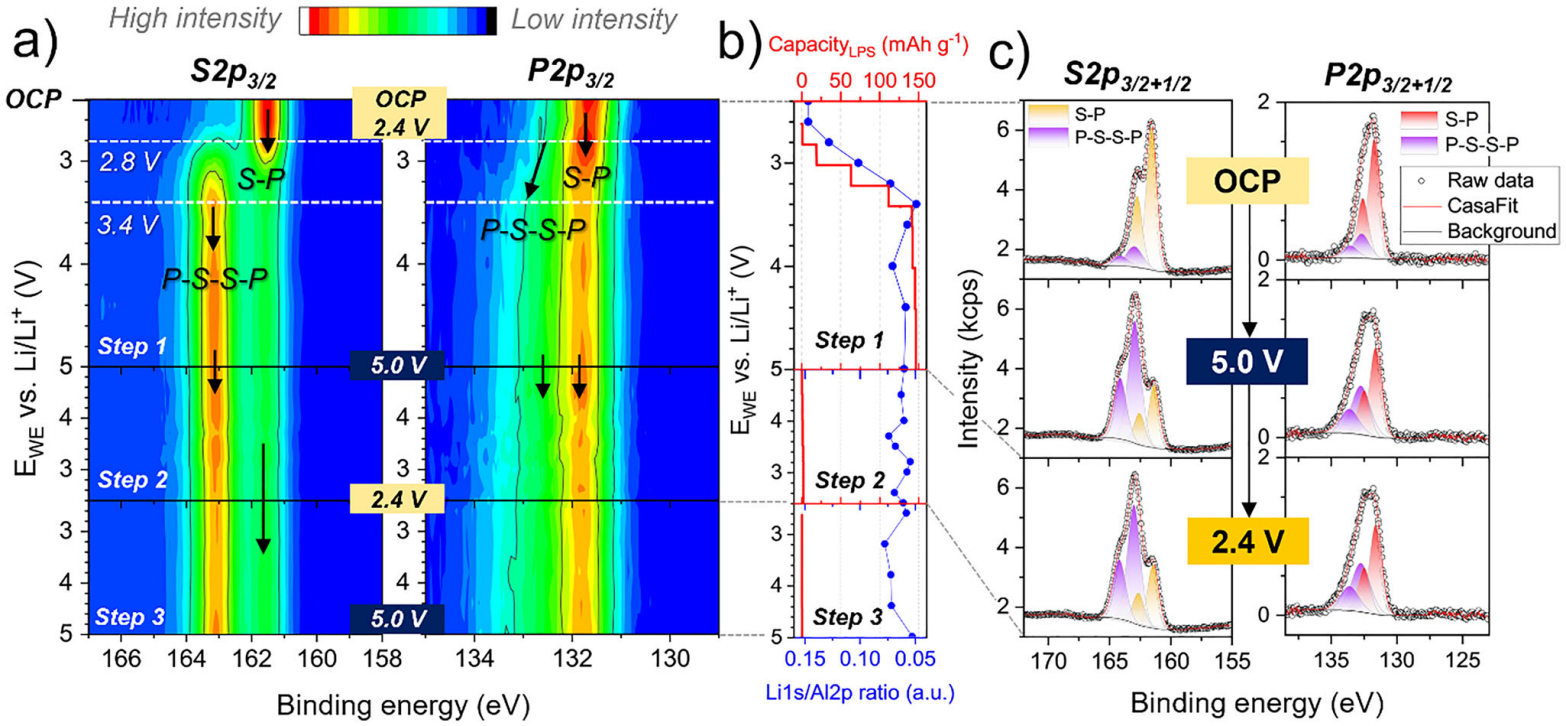
Operando XPS measurements in the high voltage range (steps 1, 2, and 3) of the LPS:VGCF WE. a) Color‐coded intensity maps of the S2p_3/2_ and P2p_3/2_ core spectra evolution at various potentials. b) The corresponding specific capacity of oxidized LPS, together with the Li1s/Al2p ratio calculated for each potentiostatic step. c) Selection of fitted S2p_3/2+1/2_ and P2p_3/2+1/2_ core level spectra at selected potentials, including the raw data, attributed compounds and fitted components. More spectra are shown in Figure S4, Supporting Information.

In **Figure** **2**a, we report the color‐coded intensity map of all the S2p_3/2_ and P2p_3/2_ core level spectra evolution in the high voltage range (steps 1 to 3). The 1/2 spin‐orbit components were mathematically subtracted, permitting better recognition of the newly formed compounds, their corresponding FWHM and intensity change, which supports the accurate identification of the byproduct species. A specimen of the fitted S2p and P2p with both 3/2 and 1/2 spin‐orbits is presented in Figure 2c and Figure S4, Supporting Information (see Table S1, Supporting Information with the corresponding fitting parameters). Additionally, all the C1s and O1s spectra evolving between steps 1 and 3 are presented in Figure S5, Supporting Information.

Based on the color‐coded intensity map and the fitted spectra, at OCP the most intense peaks in the S2p_3/2_ and the P2p_3/2_ are observed at a binding energy (BE) of 161.5 eV and 131.7 eV, respectively. They originate from the PS_4_^3‐^ local environment within the LPS and are attributed to S─P bonds. It is worth mentioning that decomposed LPS in the form of bridging sulfur (P‐S‐S‐P) species is detected already at OCP located in the S2p and P2p spectra at 162.9 eV^[^ ^62^ ^]^ and at 132.6 eV, respectively, highlighted in purple in Figure 2c and Figure S4, Supporting Information. Those impurities are also observed on the as‐synthesized LPS powder, but their amount increases after the ball milling with VGCF from 8.5 at% to 16 at% (see Figure S6 and Table S2 with Equation S1, Supporting Information for further details). In addition to the bridging sulfur, oxygen, and carbon impurities are also detected on the as‐synthesized LPS surface, confirmed by the O1s and C1s peaks located at 531.8 eV and 284.8 eV (Figure S6, Supporting Information). However, no signs of sulfates/sulfites and phosphates/phosphites are observed, as their amounts are below the detection limit. Furthermore, in Figure 2b we present the amount of Li evolution within the LPS along the cycling in steps 1 to 3, by plotting the ratio between the fitted area below the Li1s peak of LPS and the area below the electrochemically inactive Al2p metallic Al peak of the Al‐mesh CC. The ratio Li1s/Al2p is also correlated with the extracted specific capacity of the delithiated and lithiated LPS during its oxidation and reduction process, respectively. A specimen of the Al2p and Li1s fitted spectra at different potentials is reported in Figure S4c, Supporting Information.

*In the first charge (step 1) between 2.4 and 5.0 V*, the S2p_3/2_ component at 162.9 eV, attributed to polysulfide formation (P‐S‐S‐P), begins to evolve at 2.8 V (Figure 2a), accompanied by an intensity drop for the S─P (PS_4_^3‐^) component. A similar trend is observed simultaneously for the P2p_3/2_, obvious from the peak broadening at 132.6 eV assigned to the oxidized LPS and a drop in the S─P component intensity. The formation of P‐S‐S‐P species indicative of LPS oxidation byproducts, continues to progressively increase in both S2p and P2p peaks with the increasing potentials, reaching a maximum intensity at 3.4 V. Beyond this voltage, up to 5.0 V, no further evolution in the P‐S‐S‐P intensity are observed in either the S2p or P2p peaks, marking the completion of the LPS oxidation process. This result is in good agreement with the electrochemical CV in Figure 1c, where the LPS oxidative current drops sharply between 3.1 and 3.5 V, indicating the end of the oxidation reaction. Furthermore, the P‐S‐S‐P evolution and the CV behavior align with the low oxidative current density observed in the *operando* XPS cell after 3.4 V, which rapidly stabilizes below 0.01 mA cm^−2^ (Figure S3a, Supporting Information). This indicates that only a small amount of lithium is extracted, as 96 % of the maximum specific capacity of 149 mAh g^−1^ is already reached at 3.4 V. This finding is supported by the progressive decrease in the Li1s core level signal, which reaches its minimum intensity at 3.4 V (Figure 2b and Figure S4, Supporting Information).

*When reducing the potential to 2.4 V (step 2) and oxidizing again to 5.0 V (step 3)*, no significant intensity evolution of the P‐S‐S‐P oxidized compounds is observed. This is also in accordance with the CV in Figure 1c, where a minor redox activity is detected with an extracted accumulated capacity of less than 2 mAh g^−1^ (Figure S3b,c, Supporting Information). This is backed by the absence of any change in the Li1s/Al2p ratio in Figure 2b. The fitted S2p and P2p peaks displayed in Figure 2c and Figure S4, Supporting Information provide a better visualization of the S─P and P‐S‐S‐P components and allow precise comparison of their absolute intensities.

**Figure 3:**
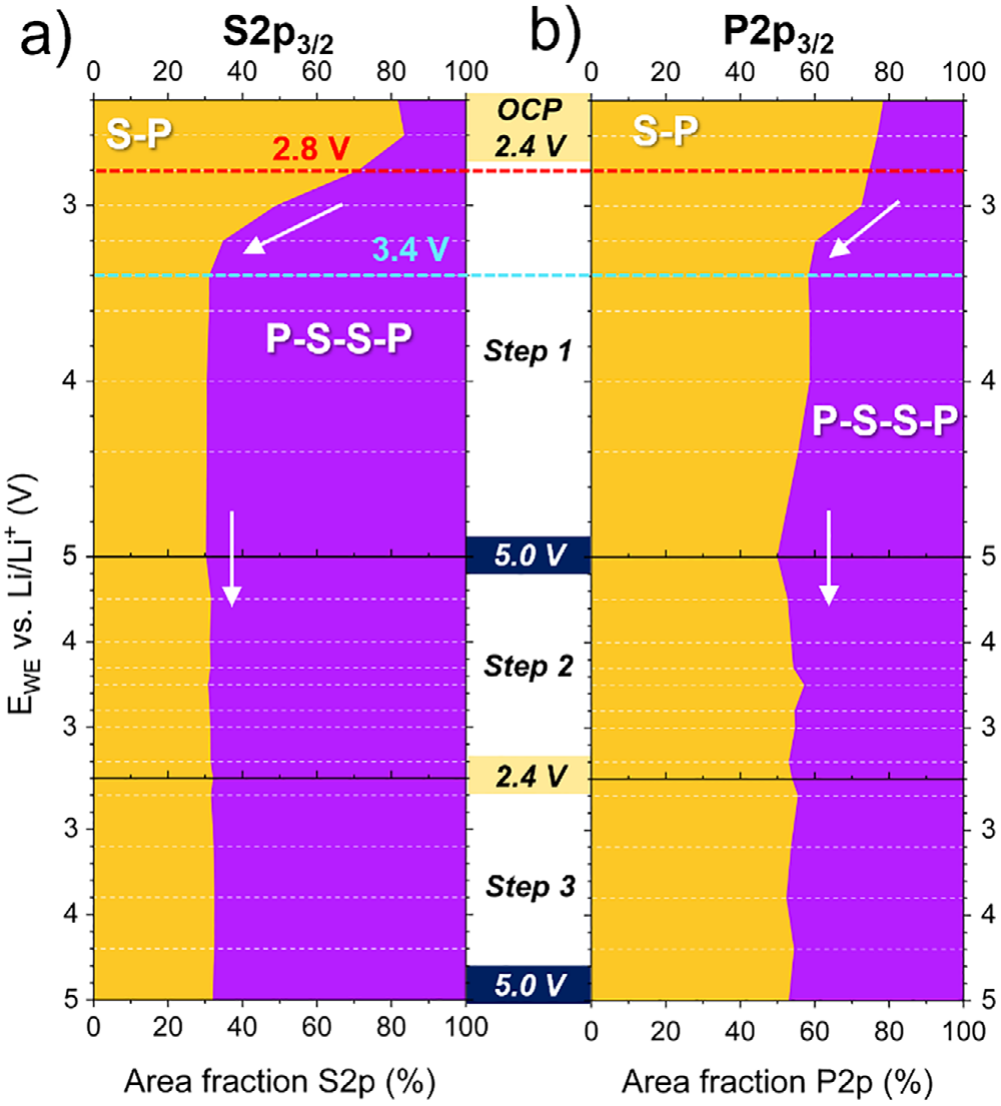
Area fraction in the high voltage range (steps 1, 2, and 3) calculated for S─P and P‐S‐S‐P fitted components within the a) S2p_3/2_ and b) P2p_3/2_ core level spectra. Dashed horizontal lines indicate the potential at which the corresponding XPS spectra were taken. Areas between the white lines are extrapolated and do not represent additional empirical values.

Furthermore, **Figure** **3** depicts the area fraction of the S─P and P‐S‐S‐P fitted components within the S2p and P2p core levels. At OCP, roughly ≈80 % of S─P non‐oxidized species and ≈20 % of P‐S‐S‐P oxidized species are measured in both S2p and P2p core level spectra (Table S2, Supporting Information). Along the first charge to 5.0 V (step 1), the amount of P‐S‐S‐P increases in the S2p core spectra to 70 %, with a decreasing amount of S─P to ≈30 % (Figure 3a). This area fraction remains unchanged after 3.4 V, and for the subsequent first discharge (step 2) and second charge (step 3). The phosphorus (Figure 3b) follows a similar trend, but the area fraction of the P‐S‐S‐P in the P2p core spectra increases only to 50 % at 3.4 V. This can be explained by its lower sensitivity to the local bonding environment changes with respect to the sulfur, as the P atoms keep the same first local bonding environment with S in the PS_4_ tetrahedra. Accordingly, the oxidized LPS byproducts have a smaller chemical shift in the P2p core level as the oxidation of the LPS takes place via the bridging sulfur between the PS_4_^3‐^ units, meaning that the P is affected by the second nearest neighbor atomic changes.

*In summary*, in the high voltage range, the LPS oxidation byproducts at the interface with the VGCF are principally polysulfides formed via the bridging sulfur (P‐S‐S‐P) between the PS_4_^3‐^ units, which is highly depleted in Li. The LPS oxidation process starts at 2.8 V and ends at 3.4 V, while no further interphase evolution is observed afterward up to 5.0 V (step 1). Between 2.4 and 5.0 V, the bridging sulfur is not reversible and the interphase remains stable even after further cycles (steps 2 and 3). Comparing to literature, we have summarized the BE positions reported from *post mortem* experiments for S^0^, bridging sulfur (‐S‐S‐), PS_4_^3‐^ (S─P) and Li_2_S in Table S3, Supporting Information. Unlike the commonly reported species assignment upon oxidation, we did not detect elemental sulfur (S^0^) in the entire high voltage region as described in Equation 1, as the S^0^ component for oxidized LPS is supposed to exhibit a BE difference ΔBE(S^0^‐PS_4_^3‐^) ≥ 2 eV against PS_4_^3‐^. In our case, we observe just one component with ΔBE = 1.4 eV against PS_4_^3‐^, which is in agreement with P‐S‐S‐P component reported in literature with ΔBE values between 1.2 and 1.8 eV. Last, the O1s and C1s remain unchanged (see Figure S5, Supporting Information), confirming that the only species evolving at the interface between the LPS and VGCF are the polysulfides and do not cover the surface of the VGCF.

#### 2.2.3 Operando XPS in the Low Voltage Range to 0.6 and −0.05 V

**Figure 4:**
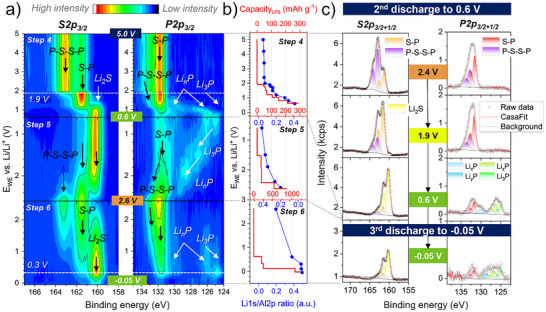
Operando XPS in the low voltage range (steps 4, 5, and 6) of the LPS/VGCF WE composite. a) Color maps for the S2p_3/2_ and P2p_3/2_ core level spectra evolution. b) The corresponding specific capacity of reduced LPS, together with the Li1s/Al2p ratio calculated for each potentiostatic step. c) A selection of fitted S2p_3/2+1/2_ and P2p_3/2+1/2_ core level spectra at specific potentials, including the raw data, attributed compounds, and fitted components. Voltage‐dependent shifts of binding energies are corrected. Additional spectra are shown in the Figure S8, Supporting Information.

**Figure 5:**
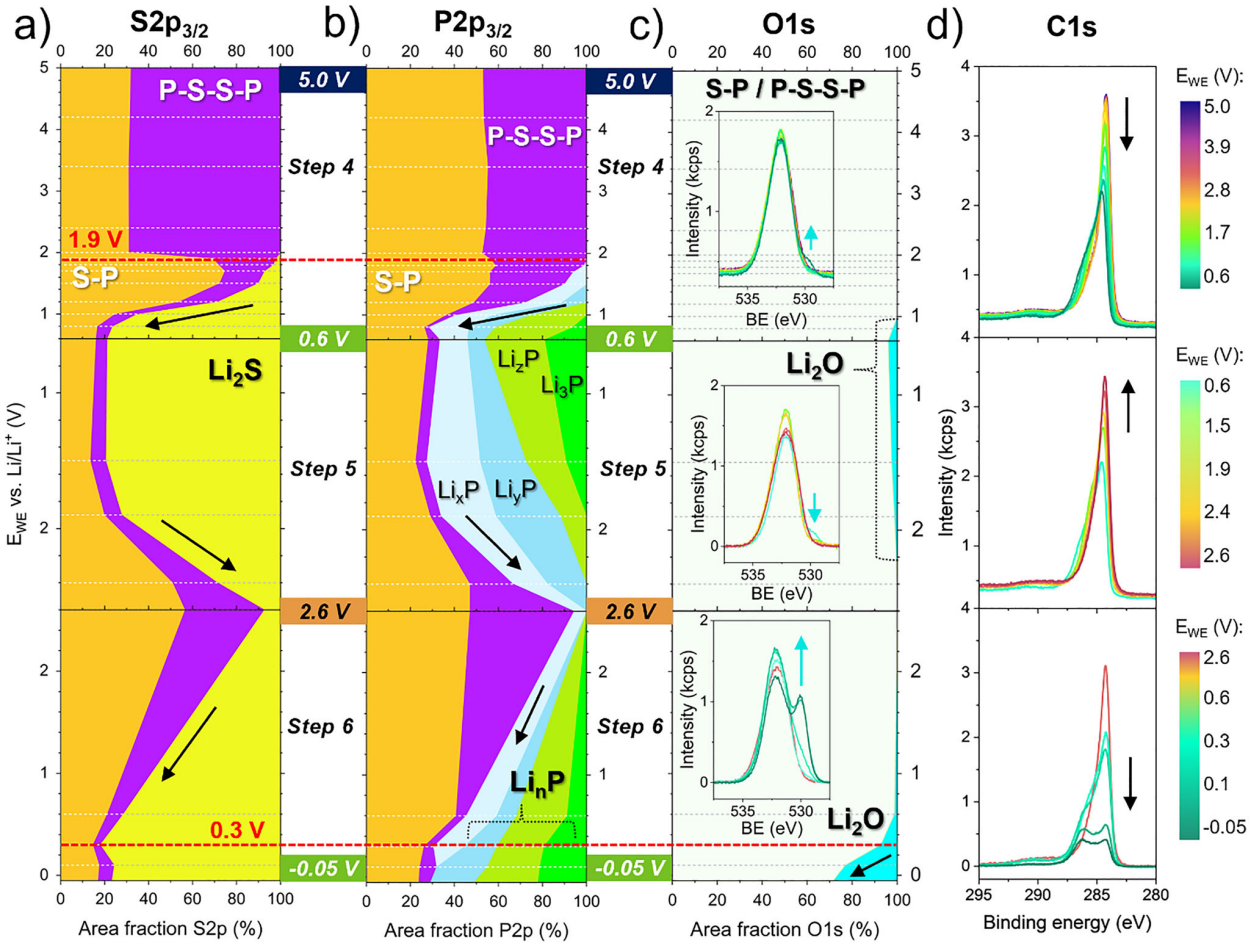
Area fraction in the low voltage range (steps 4, 5, and 6) calculated for S─P, P‐S‐S‐P, Li_2_S, Li_n_P (0 ≤ n ≤ 3), and Li_2_O fitted components within the a) S2p_3/2_, b) P2p_3/2_, and c) O1s core level spectra. Dashed horizontal lines indicate the potential at which the corresponding XPS spectra were taken. Areas between the dashed lines are extrapolated and do not represent additional empirical values. In (c), the corresponding O1s core level spectra are provided as an inset. d) C1s core level spectra evolution over the entire low voltage range. Voltage‐dependent shifts of binding energies in (c) and (d) are corrected.

*In the second discharge from 5.0 V until 0.6 V (step 4)*, the S2p_3/2_ and P2p_3/2_ evolution reported in **Figure** **4**a shows significant changes as soon as the WE entered more reductive potentials. Below 2.0 V, the polysulfide oxidized species are substantially reversible, as they are reduced back to the PS_4_^3‐^ tetrahedral environment of LPS. The P‐S‐S‐P components, located at 162.9 eV in the S2p and at 132.6 eV in the P2p spectra, drop in intensities while the S─P (PS_4_^3‐^) components at 161.5 eV and at 131.7 eV increase (Figure 4a,c). The conversion reaction from the bridging sulfur to PS_4_^3‐^ reaches its maximum at 1.7 V (see **Figure** **5**a), with a reversible capacity of 59 mAh g^−1^ in Figure S7a, Supporting Information, endorsed by the rise of the Li amount monitored by the Li1s/Al2p ratio (Figure 4b and Figure S8c, Supporting Information). This conversion reaction can be precisely assigned to the redox peak in the CV located at 1.6 V during the negative sweep (Figure 1b).

Interestingly, 1.9 V is also the onset potential for the lithium sulfide (Li_2_S) formation, where a tiny component appears ≈160.0 eV in the S2p (Tables S4 and S5, Supporting Information, and the fitted S2p peak in Figure 4c and Figure S8a, Supporting Information). Subsequently, below 1.9 V, both the polysulfide and the PS_4_^3‐^ components are simultaneously converted to Li_2_S. This conversion is evidenced by a significant intensity drop of both P‐S‐S‐P (162.9 eV) and S─P (161.5 eV) components, accompanied by a strong increase of the Li_2_S component at 160.0 eV in the S2p core levels (Figure 4a,c, and Figure S8a, Supporting Information).

In addition to the Li_2_S formation, lithium phosphides (Li_n_P, 0 ≤ n ≤ 3) are also concurrently evolving, where various lithiation phases are formed starting at 1.8 V, visible through additional components appearing between 130.2 eV and 124.6 eV in the P2p core levels (Figure 4a,c and Figure S8b, Supporting Information). The multitude of lithium phosphide compounds evolving in the P2p spectra are fitted in different components as shown in Figure 4c, depending on the amount of lithium enrichment with 0 ≤ n ≤ 3. We have labeled Li_x_P, Li_y_P, Li_z_P and Li_3_P the components evolving at 130.2, 128.1, 126.7, and 124.6 eV BE, respectively, as summarized in Table S6, Supporting Information. Following the electronegativity of phosphorus and lithium elements, we attributed the Li_x_P (x = 0) component with the highest BE to elemental phosphorus (P^0^), with an onset potential of 1.8 V, which is often reported to be at ≈129.6 eV.^[^ ^44^ ^]^ We assigned the Li_y_P (y = 1) and Li_z_P (z = 2) to the intermediate lithiation phases (P^1‐^) and (P^2‐^) with the onset potentials of 1.2 and 1.1 V, respectively. Last, reducing the cell potential beyond 0.8 V, a strongly reduced (P^3‐^) compound evolves at the lowest detected BE position of 124.6 eV, which is attributed to the formation of Li_3_P.^[^ ^30^, ^63^ ^]^

Figure 5a,b display the area fraction evolution of the various fitted components within the S2p and P2p core level spectra. We can precisely identify the formation onset potential of each species and monitor their intensity evolution. It provides a dynamic view of the conversion reactions in step 4, starting with the conversion of P‐S‐S‐P to PS_4_^3‐^ within the narrow potential window of 1.9–1.7 V, followed immediately by the second conversion reaction within 1.9–0.6 V where both P‐S‐S‐P and PS_4_^3‐^ are progressively reduced to Li_2_S and Li_n_P. At 0.6 V, the reduced LPS is dominated mainly by Li_2_S (79 %) in S2p and Li_n_P (67 %) in P2p, with only a small amount of remaining PS_4_^3‐^ (16 %) and polysulfides (5 %) in Figure 5a. The latter reaction provided an additional specific capacity of ≈246 mAh g^−1^, supported by the continuous rise of the Li amount monitored by the Li1s/Al2p ratio (Figure 4b and Figure S8c, Supporting Information). In correlation with the CV in Figure 1b, we can accurately assign the redox peak at 0.6 V during the negative sweep to both conversion reactions Li_2_S and L_n_P.

Near the end of step 4 at 0.8 V, the Li_2_O component is detected in the O1s (Figure 5c) located at 530.0 eV which is caused either by the competitive reactions between the lithiated phosphorus and the residual oxygen in the UHV or the reduction of the residual oxygen on the VGCF surface by the lithium‐ion from the LPS (Equation 5).

(5) $$4Li^{+}+2e^{-}+O_{2}↔2Li_{2}O$$

Furthermore, unlike in the high voltage range, the C1s core spectra in the low voltage range undergo changes related to the progressive intensity drop of the main component at 284.3 eV of C─C bonds originating from the VGCF peak (Figure 5d). It correlates with the progressive intensity increase of the Li_2_S and Li_n_P components, confirming their growth on the VGCF surface.

*During the third charge from 0.6 to 2.6 V (step 5)*, the reversibility of the reduced species Li_2_S, Li_n_P, and Li_2_O is investigated (Figures 4a and 5a, and Figure S8, Supporting Information). Between 0.6 and 1.5 V, no significant changes are observed in the S2p spectra, confirming that Li_2_S is stable within this voltage range. In contrast, major changes are observed in the P2p spectra, where the intensity of the Li_3_P and the Li_2_P components drops progressively, while the intensity of the LiP and P^0^ increases. This is induced by the de‐alloying process of the Li_3_P to P^0^ going through all the intermediate phases Li_2_P and LiP,^[^ ^63^ ^]^ which can be clearly attributed to the redox peak at 1.3 V during the positive sweep in the CV of Figure 1b. Subsequently, at higher potential between 1.9 and 2.6 V, the Li_2_S becomes active, and a reversible conversion of both Li_2_S and Li_n_P to PS_4_^3‐^ is observed (Figure 4a). It is confirmed by the intensity drop of the Li_2_S component at 160.0 eV and the increase of the S─P component at 161.5 eV in the S2p spectra (Figure 5a). The same behavior is observed in the P2p spectra, where the intensity of the Li_n_P components (130.2–124.6 eV) drops and the S─P component at 131.7 eV increases. As oxidation progresses, the Li_3_P and Li_2_P are not detectable anymore at 1.9 and 2.4 V, respectively, as they have been converted to P^0^ and LiP (Figure 5a). Above 2.4 V, a simultaneous reaction between the formation of reversible PS_4_^3‐^ and its oxidation to polysulfides is evidenced. At 2.6 V, the polysulfides are well pronounced in both S2p and P2p spectra, confirmed by the increase of the P‐S‐S‐P component at 162.9 and 132.6 eV, respectively. Additionally, a small fraction of Li_2_S and P^0^ remains in the WE, suggesting that these compounds have undergone predominantly reversible conversion. A reversible capacity of 206 mAh g^−1^ (Figure S7b, Supporting Information) is obtained at 2.6 V for step 5, which is again in good agreement with the decreasing Li1s/Al2p peak ratio over the delithiation process (Figure 4b). For those last conversion reaction from Li_2_S to PS_4_^3‐^ and P‐S‐S‐P, the CV in Figure 1b is unable to resolve the two redox reactions, and it is just showing one single broad redox peak centered at 2.7 V. Last, the reversible oxidation reaction was also observed for the Li_2_O species, which fully vanished from the WE between 1.9 and 2.6 V, confirmed by the disappearance of the Li_2_O component located at 530.0 eV in the O1s core level spectra (Figure 5c). The C1s core level also shows considerable changes in step 5, as the intensity of the VGCF component at 284.3 eV increased progressively and reached its maximum at 2.6 V in correlation with the oxidation of the Li_2_S and Li_n_P species, which we believe to be covering the VGCF.

*The third discharge from 2.6 to −0.05 V (step 6)* aims to investigate the effects of significantly lower potentials below 0.6 V on the formation and evolution of Li_2_S, Li_n_P, and Li_2_O species, as well as the extent of their reactivity. Additionally, this step explores the possibility for detecting lithium plating below 0.0 V. A quick discharge in the voltage from 2.6 to 0.6 V is performed (Figures 4a and 5a), during which the P‐S‐S‐P and PS_4_^3‐^ are reversibly reduced to Li_2_S and Li_n_P, as confirmed by the fitted S2p and P2p core level spectra (Figure S8a,b). A similar amount of Li_2_S and Li_n_P as in step 4 is reformed at 0.6 V, based on the area fraction calculated in Figure 5a for S─P (21 %), P‐S‐S‐P (7 %), Li_2_S (72 %) and in Figure 5b for Li_n_P (55 %), providing a specific capacity of 205 mAh g^−1^ in step 6 (Figure S7c, Supporting Information and Figure 4b). The reversibility of the redox reaction is also confirmed by the reversible amount of Li, measured from the Li1s/Al2p ratio of 0.41 and 0.38 at 0.6 V in step 4 and step 6, respectively. Moreover, the formation of Li_2_O species is repeated (Figure 5c), and the intensity drop of the VGCF component at 284.3 eV (Figure 5d) is comparable to step 4.

Further reduction beyond 0.3 to −0.05 V does not show additional species evolving in the S2p and P2p core spectra but indicates a strong drop in the overall intensity, as shown in Figure 4a,c, and Figure S8a,b, Supporting Information. However, a strong increase in the Li_2_O component located at 530.0 eV in the O1s (Figure 5c) is observed, accompanied by a strong intensity decrease in the VGCF C1s component at 284.3 eV (Figure 5d). This certifies that the reduced byproducts of LPS and Li_2_O layers cover the LPS and VGCF surface.

Given the offset in the discharge capacity evolution and the changes in Li1s/Al2p ratio in Figure 4b for step 6, there seems to be an additional reaction taking place, with a strong consumption of charges at 0.2 V reaching a specific capacity of 1090 mAh g^−1^ (Figure 4b and Figure S7c, Supporting Information). This can be explained by two parallel reactions taking place at highly reductive potentials. First, the Al CC‐mesh is not stable at such low potentials, starting to alloy with lithium ≈0.3 V upon reduction,^[^ ^64^ ^]^ clearly visible in the CV data of the *operando* cell at low potentials (Figure S2, Supporting Information). This reaction solely takes place below the Al‐mesh, as there is no sign of Li‐Al alloy in the XPS Li1s and Al2p core level spectra (Figure S8c, Supporting Information). Further, between 0.1 and −0.05 V, we assume that metallic Li^0^ would have also plated on the VGCF, however, no metallic lithium has been detected in the Li1s core level spectra, but rather Li_2_O has formed.

Additional in‐plane *post mortem* SEM‐EDX analysis performed on the surface of the WE composite (Figure S9, Supporting Information) suggests that the VGCF and the composite surface were covered with oxygen‐rich species at −0.05 V when compared to the SEM‐EDX images of the pristine composite WE.

*In summary*, in the low voltage range, the polysulfides formed above 2.8 V become substantially reversible below 2.0 V. The polysulfides convert to PS_4_^3‐^ between 1.9 and 1.7 V, while the formation of Li_2_S initiates in parallel. Additionally, Li_n_P evolves concurrently starting at 1.8 V, with various lithiation phases from P^0^ to LiP, Li_2_P and Li_3_P. By the end of the discharge to 0.6 V, the reduced LPS primarily consists of Li_2_S and Li_n_P, forming a Li‐enriched interface between the SE and VGCF. Between 0.8 and 0.6 V traces of Li_2_O are also detected, assigned to residual oxygen in the UHV chamber, which is chemically reacting with Li_n_P or electrochemically reduced on the surface of the VGCF upon contact with Li‐ions from the SE. During the charge from 0.6 to 2.6 V, the reversibility of the redox reactions is confirmed, as the Li_n_P starts the de‐alloying process immediately. However, the Li_2_S de‐alloys much later, between 1.9 and 2.6 V, and is first converted to PS_4_^3‐^ followed by an oxidation to polysulfides with bridging sulfur. Furthermore, the Li_2_O was also fully oxidized by the end of the charge. Finally, below 0.1 V, the strong increase of Li_2_O in the O1s spectra indicates the plating of lithium on the surface and its immediate reaction with residual oxygen in the UHV.

Unlike other reported paper performing in situ*/virtual operando* XPS or Auger electron spectroscopy,^[^ ^21^ ^]^ by plating Li^0^ in situ with an electron gun on the sulfide SE surface, we argue that Li_2_O formation does not stem from oxygen impurities originating from the pristine SE lattice, as we do not detect sulfate/sulfite or phosphate in both S2p and P2p spectra.

#### 2.2.4 Electrical Properties of the Interphase

Besides the identification and quantification of the various LPS electrochemically reduced and oxidized chemical species evolution, *operando* XPS bears great potential to study their corresponding surface electrical properties evolution in real‐time. This has been widely explored for different electrified gas‐/liquid‐solid interfaces,^[^ ^50^, ^51^, ^54^, ^65^, ^66^, ^67^ ^]^ and for the dynamic changes in the surface potential at solid‐solid interfaces.^[^ ^46^, ^55^ ^]^ As a matter of fact, when grounding the WE to the analyzer, the apparent BE shift of the core‐level spectra at a defined applied external voltage (V_cell_) is given by:

(6) $$\Delta BE=BE\left(V\right)-BE\left(OCP\right)=-e\left(\Delta V_{cell}-\Delta V_{S}\right)$$

With ΔV_cell_ = V_cell_ (V) ‐ OCP and ΔV_S_ is the local variation in surface potential of the respective compound detected in their elemental core level spectra, defined as ΔV_S_ = V_S_ (V) – V_S_ (OCP).^[^ ^53^ ^]^ Following Equation 6, three different scenarios can be presented on the composite WE. First, for the electrically conductive compounds their corresponding elemental core levels will capture the full applied potential with V_S_ = V_cell_, such as in the case of the VGCF conductive carbon. Thus, the C1s originating from the VGCF will not shift with the applied potential, hence ΔBE = 0. Second, for the opposite situation, the electrically insulating compounds, such as the LPS solid electrolyte, the V_S_ = 0. Consequently, their corresponding core levels, like S2p, P2p or Li1s, should shift linearly with the applied potential as ΔBE = ‐ e*ΔV_cell_. Third, for intermediate situations 0 < V_S_ < V_cell_ applies, as in semiconducting compounds or when an electrical charge induces local surface overpotential changes. In this case, ΔBE can be described as ΔBE = ‐e*η_S_, where η_S_ = ΔV_cell_ ‐ ΔV_S_, which we use as a measure of the potential change across the surface and interface for different particles.

**Figure 6:**
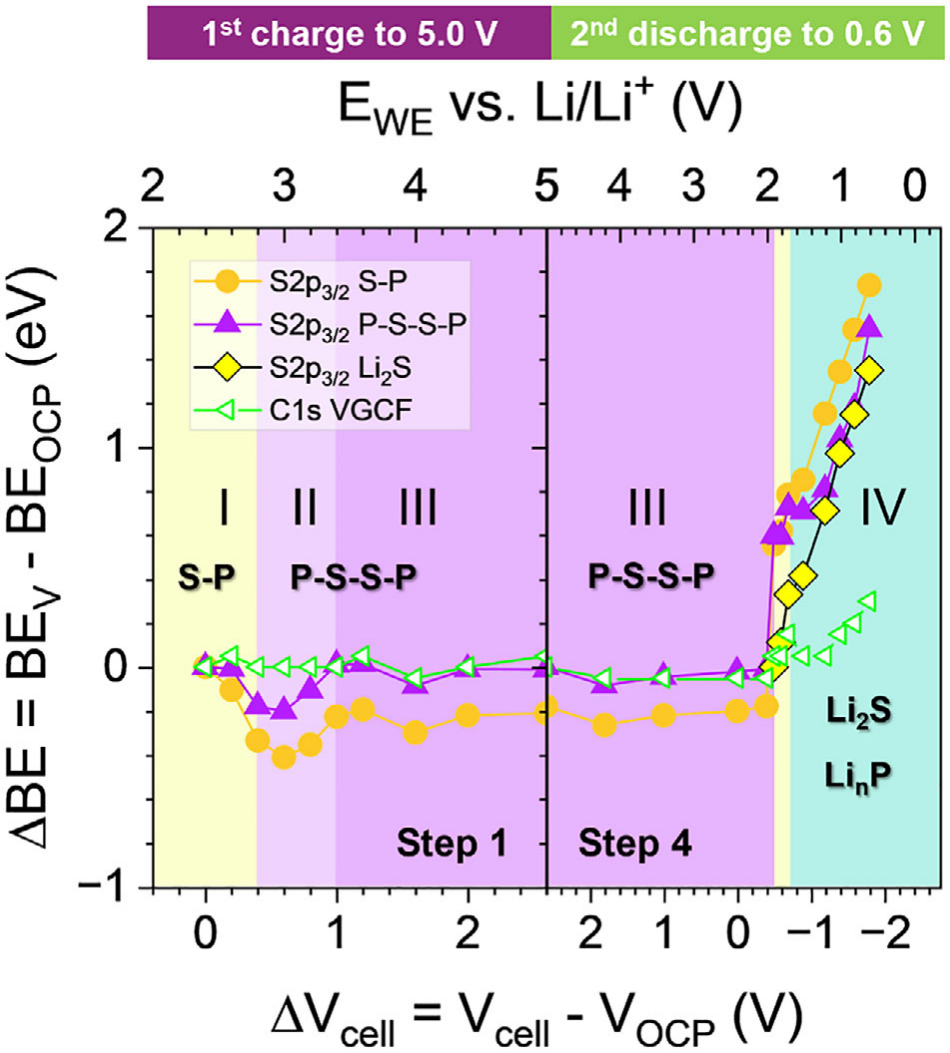
S2p_3/2_ binding energy (BE) shift evolution for the S─P, P‐S‐S‐P, and Li_2_S fitted components, and for the C1s peak from VGCF during the oxidation to 5.0 V (step 1) and the reduction to 0.6 V (step 4). Sequences I, II, III, and IV indicate different surface electrical behaviors. Each sequence is connected in a color code to the predominant species that was formed at the specific voltages.

**Figure** **6** displays the difference in BE peak position at different cell voltages with respect to its position at OCP, calculated as ΔBE for each species. We focused first on the S2p_3/2_ peaks BE evolution, which originate from the following species: non‐oxidized LPS (S─P), oxidized LPS polysulfides (P‐S‐S‐P), and reduced LPS (Li_2_S), as well as the C1s peak at 284.3 eV of the C─C bonds in VGCF. Both high and low voltage ranges are investigated for the first charge up to 5.0 V (step 1) and the second discharge to 0.6 V (step 4), with further intermediate steps examined and reported in Figures S10 and S11, Supporting Information.

*High voltage range to 5.0 V*: Herein, the BE peak shift of the VGCF C─C bonds in the C1s core level spectra serves as a good reference for an electrically conductive compound. As expected, there is no significant shift of its initial peak position at OCP in step 1 between 2.4 and 5.0 V (Figure 6), as well as in step 2 (5.0–2.4 V) and step 3 (2.4–5.0 V) (Figure S10, Supporting Information). Following the BE peak shifts evolution of the S─P and P‐S‐S‐P components in the S2p_3/2_ core level, we identified three distinct behaviors in step 1. In sequence (I) between 2.4 (OCP) and 3.0 V, both species shifted linearly with the applied voltage ΔBE = ‐ e*ΔV_cell_, a characteristic behavior for electrically non‐conductive species, aligning well with the reported low electrical conductivity values of LPS in literature (see Table S7, Supporting Information).^[^ ^68^ ^]^ Within sequence (II) between 3.0 and 3.4 V, the BE shift becomes non‐linear with the applied voltage and increases progressively to ΔBE = 0 at 3.4 V, characteristic of electrically conductive species. Such a transition in the electrical properties of the S─P and P‐S‐S‐P coincides well with the onset oxidation potential of LPS to form polysulfides at 2.8 V and with the end of the oxidation reaction at 3.4 V (Figure 3). After 3.4 V in sequence (III) no further S2p BE shifts are observed for both S─P and P‐S‐S‐P components in the high voltage range (ΔBE = 0), even during the first discharge (step 2) and the second charge (step 3) (Figure S10a, Supporting Information), demonstrating that the formed polysulfide (P‐S‐S‐P) at the interface with the VGCF is electrically conductive. The same behavior is also observed for the P2p core level of the S─P and P‐S‐S‐P components (Figure S10b, Supporting Information).

*Low voltage range to 0.6 and −0.05 V*: Likewise, in sequence (III) during step 4 from 5.0 to 2.0 V (Figure 6 and Figure S11, Supporting Information), no BE shift is noticed for the S2p, P2p, and C1s core levels, which is anticipated as no chemical alteration occurs at the interface in this voltage range. The amount of the S─P and P‐S‐S‐P remains the same (Figure 5a), thus, no change in the electrical properties is expected at the interface. However, major changes appeared from 1.9 to 0.6 V in sequence (IV), where a sudden linear peak shift of all S2p and P2p components with respect to ΔV_Cell_ appeared (ΔBE = +1 eV V^−1^), demonstrating that the electrical properties of the interface switch from being an electrical conductor to an electrical non‐conductor. Interestingly, it coincides with the reversible conversion of the P‐S‐S‐P polysulfide to PS_4_^3‐^ between 1.9 and 1.7 V, followed by the conversion of LPS to Li_2_S and Li_n_P between 1.7 and 0.6 V (Figure 5a). This indicates a strong electrically insulating character of the PS_4_^3‐^ and Li_2_S/Li_n_P, as supported by electrical conductivities reported in literature (see Table S7, Supporting Information). Concurrently, the BE of C1s assigned to VGCF also starts to shift slightly below 1.9 V, reaching at 0.6 V a ΔBE = 0.3 eV V^−1^. It proves the overpotential increase across the VGCF surface in correlation with the intensity drop reported for the C1s core level spectra (Figure 5d), assigned to the Li_2_S/Li_n_P and Li_2_O coverage of the VGCF surface, which impedes the electric transport across the interface. Regarding step 5 (0.6 to 2.6 V) and step 6 (2.6 to −0.05 V) in Figure S11, Supporting Information, no major change occurs in the linear BE shift of the various components in the S2p and P2p, meaning that in those voltage ranges the interface remains electrically non‐conductive, which is consistent with the interface chemistry rich in Li_2_S, Li_n_P, and Li_2_O (Figure 5a).

### 2.3 Operando Stack Pressure Monitoring

**Figure 7:**
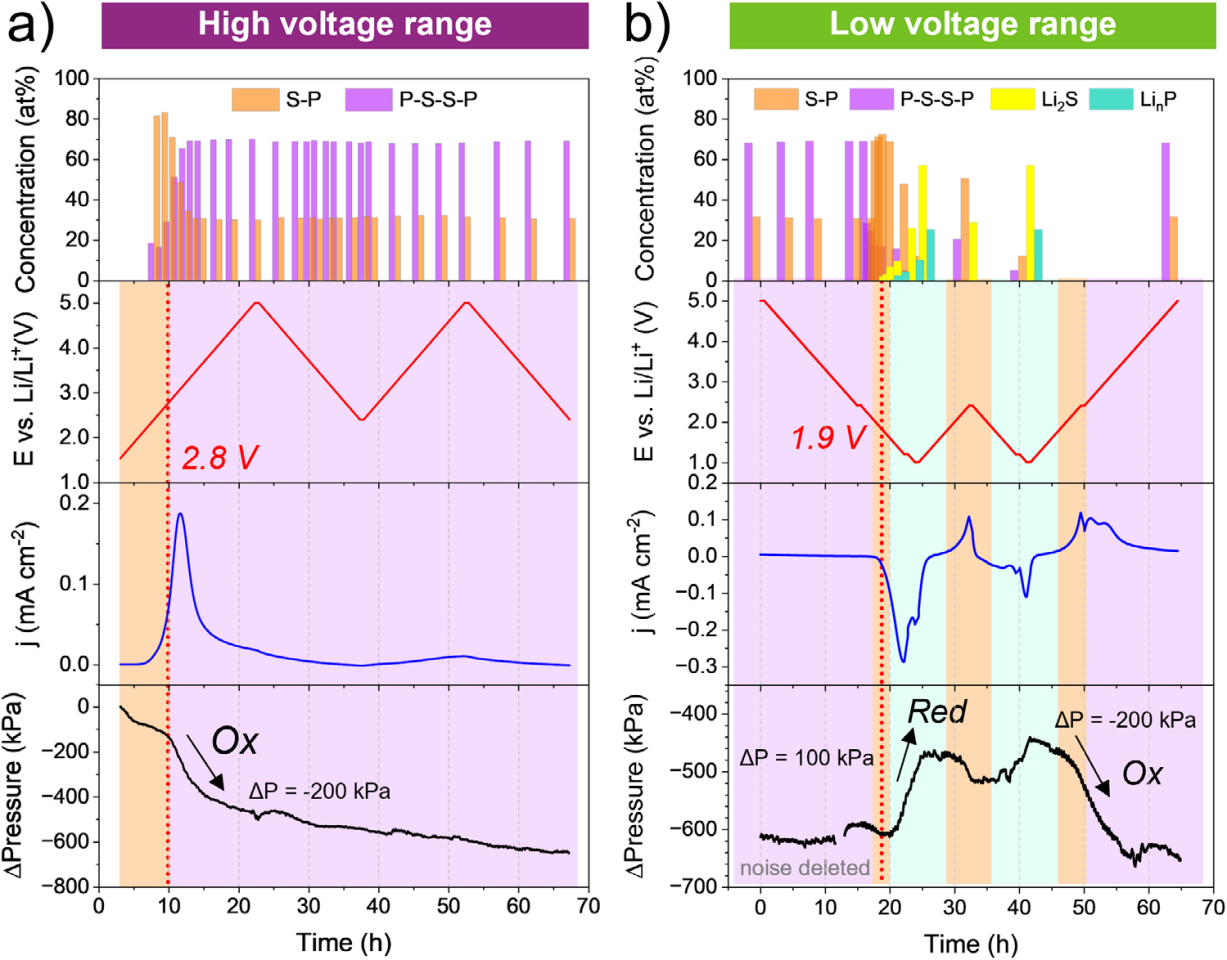
Operando stack pressure monitoring for LTO|LPS|LPS:VGCF ASSB during the CV cycling, a) in the high voltage range (1.5–5.0 V) and b) extended to the low voltage range (5.0–1.0–2.4 V) and finished with an oxidation from 2.4 to 5.0 V. The background color highlights the predominant species evolving, with S─P (orange), P‐S‐S‐P (purple), Li_2_S (yellow), Li_n_P (light green).

The full picture of the LPS interface chemical evolution with VGCF would not be completed without its accurate correlation with the interface mechanical integrity. To advance the understanding of the interface mechanical stability, we monitored the cell pressure evolution in real‐time during cycling as a direct probe of the volume change inside the WE. A model ASSB stack was designed for this purpose, composed of ball milled (80 wt% LPS and 20 wt% VGCF) WE cycled versus Li_4_Ti_5_O_12_ (LTO) as a counter electrode (CE) composite. LTO is used as CE active material, since it does not undergo any volume change during the Li insertion/extraction processes.^[^ ^69^ ^]^ Therefore, any cell pressure evolution is directly linked to the volume change caused by the LPS oxidation/reduction byproducts, as both the VGCF and LTO undergo no volume change. In **Figure** **7**, we present the *operando* cell pressure evolution during the CV cycling of the LTO|LPS|LPS:VGCF stack in the (i) high voltage range where two cycles are performed within the cut‐off voltage (1.5–5.0 V) (Figure 7a), (ii) low voltage range where two cycles are performed within the voltage limits (5.0–1.0–2.4 V) and (iii) finished with oxidation from 2.4 to 5.0 V (Figure 7b). Lower potentials than 1.0 V had to be excluded to not exceed the Li‐reservoir and stable redox potential of the CE. The cell pressure evolution is simultaneously correlated with the atomic % concentration calculated in Table S2, Supporting Information for the same potentials of the S─P, P‐S‐S‐P, Li_2_S, and Li_n_P species.

In the high voltage range, during the first oxidation to 5.0 V, we observe a −200 kPa drop in the cell pressure between 2.8 and 3.4 V, which correlates perfectly with the LPS electrochemical oxidation peak in the CV curve and with the onset and the end of the LPS conversion reaction to polysulfides (P‐S‐S‐P). We attribute the pressure drop to the loss of lithium from the oxidized LPS at the interface with VGCF, leading to a Li‐depleted interface and a volume shrinkage of the polysulfides as the oxidized byproducts. Such a volume change is typical in conversion materials and causes contact loss between the LPS and VGCF at the interface. Assuming full conversion of LPS to P‐S‐S‐P (Li_4_P_2_S_8_), a volume decrease of −8.7 % is theoretically predicted from our calculations in Equation S2, Supporting Information. After 3.4 V, the cell pressure remains unchanged up to 5.0 V and even for the subsequent cycles, excluding the mechanical relaxation stemming from the cell itself, which we estimated ≈−30 kPa per 10 h, see Figure S12, Supporting Information. This is expected since no further electrolyte redox process is happening after 3.4 V and no change was observed in the interface chemistry (Figure 3).

The same exercise has been repeated for the low voltage range during the CV cycling from 5.0 to 1.0 V (Figure 7b). As anticipated, no variation in the cell pressure is noticeable between 5.0 and 1.8 V, since no chemical changes happened at the LPS/VGCF interface, which remains polysulfide (P‐S‐S‐P) rich. Interestingly, between 1.9 and 1.0 V, the cell pressure increases by 100 kPa, which correlates perfectly with the reduction peak in the CV curve and with the onset of the reversible conversion reaction from polysulfide (P‐S‐S‐P) to LPS, followed by the Li_2_S and Li_n_P formation. We attribute the pressure increase to the reversible volume expansion of the LPS and to the Li‐enriched interface with Li_2_S and Li_n_P, which aligns well with the expected volume expansion upon reduction (see Equation S3, Supporting Information).

The cell pressure variation is very small during the CV oxidation and reduction between 1.0 and 2.4 V. It remains steady between 1.0 and 2.2 V during oxidation, as no changes in the interface chemical composition occur in this voltage range, followed by a slight drop of −30 kPa between 2.2 and 2.4 V during the reversible conversion reaction of Li_2_S and Li_n_P to LPS. We could also observe the same slight cell pressure increase by 70 kPa between 2.4 and 1.0 V during the reversed conversion from LPS to Li_2_S and Li_n_P. Last, during the final CV oxidation step from 1.0 to 5.0 V, the cell pressure drops by −200 kPa between 2.4 and 3.6 V, associated with the reversible formation of a Li‐depleted interface during the oxidation of LPS to polysulfides (P‐S‐S‐P).

Those results confirm that the cell pressure variation is directly linked to the interface chemistry change and follows the reversibility of the formed byproduct species, which drastically affects the mechanical stability of the WE and the contact between the particles.

## 3 Discussion

**Figure 8:**
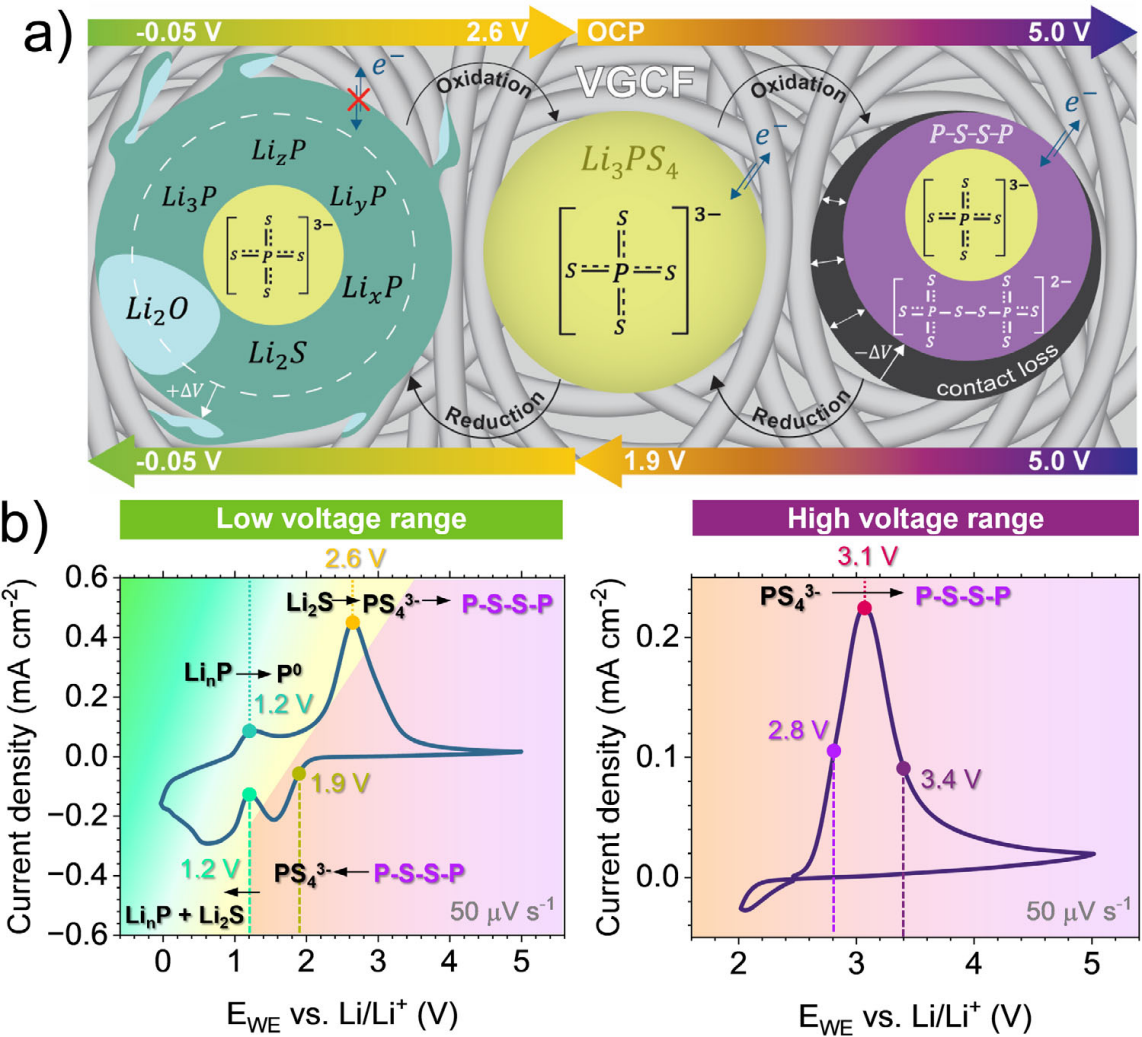
a) A summary of the electrochemical phase changes, corresponding volume changes (ΔV), and changes in the electrical (e^‐^) transport properties for both the high and the low voltage range species upon their reversible oxidation and reduction. b) CV in the low voltage and high voltage range, with the corresponding species assignment.

The sketch in **Figure** **8**a summarizes the electrochemical reactions and mechanical changes occurring at the interface between the LPS and VGCF, as well as the interface electrical properties when cycled in the high and low voltage ranges. Figure 8b complements this summary by indicating the reaction species corresponding to the redox peaks and their onset potentials in the high and low voltage range cyclic voltammetry. The oxidation of the LPS in the high voltage range (2.4 –5.0 V), imitating the cathode cycling, leads to an interphase rich in bridging sulfur (P‐S‐S‐P). The absence of elemental sulfur in our study excludes the LPS oxidation mechanism proposed in Equations 1 and 2, and endorses the mechanism proposed by *Hakari* et al.^[^ ^16^ ^]^ described as the association and dissociation of the bridging S−S bonds between the PS_4_^3‐^ units of the LPS occurring during the Li extraction−insertion processes (Figure 8a). We describe this mechanism in the revised oxidation reaction in Equation 7, as previously proposed by *Koerver* et al.^[^ ^17^ ^]^

(7) $$2Li_{3}PS_{4}\to Li_{4}P_{2}S_{8}+2Li^{+}+2e^{-}$$

Furthermore, the electrically conductive properties of the polysulfides demonstrated above are consistent with the absence of S^0^, which has a poor electrical conductivity of 10^−16^ S cm^−1^.^[^ ^70^, ^71^ ^]^ However, the polysulfide has low ionic conductivity in agreement with the high cell impedance measured at 5.0 V (Figure S13a, Supporting Information), and in accordance with a Li‐depleted interface caused by the LPS delithiation conversion reaction. Moreover, the ≈15 times increase in the cell impedance over 20 cycles (Figure S13b, Supporting Information) is directly linked with the electrically conductive character of the interface and the continuous oxidation of the LPS. Unlike what was already reported in the literature regarding the in situ passivation of the sulfide‐based SE after the first cycle,^[^ ^22^, ^23^ ^]^ our impedance results demonstrate that the LPS/VGCF interface continues evolving over the cycling, in spite of the *operando* XPS which does not show any significant changes after 3.4 V during the first charge (Figure 3). We hypothesize that this could be due to a modest progressive LPS delithiation process, in line with the low oxidative current of 0.02 mA cm^−2^ after the first charge (see inset Figure 1c), which, after all, provides 8 mAh g^−1^ of specific capacity upon second charge (see Figure S1, Supporting Information). This can result in more polysulfide formation, but below the detection limit of the XPS. Another rational explanation is the progressive local microstructural changes involving PS_4_^3‐^ tetrahedral distortion and S─S bond formation during the first charge, followed by PS_3_^‐^ motif formation and further PS_4_^3‐^ distortion during later cycles, as suggested by *Cao* et al.^[^ ^27^ ^]^ Similarly, such a process will not show any changes in the XPS peaks.

It is worth emphasizing that the working electrode volume drops (Figure 7a) during the first oxidation. As already stated above, it is induced by the LPS lithium depletion and its subsequent volume shrinkage. The shrinkage of the LPS upon oxidation is expected to cause a loss of contact between the LPS and VGCF, as illustrated in Figure 8a, contributing to an increase in cell impedance during the initial charge. However, this effect is likely to be negligible in subsequent cycles, as changes in the working electrode volume could not be detected after the first charge (Figure 7a).

All electro‐chemo‐mechanical instabilities inherent to the oxidation of LPS will also occur when LPS is cycled with high‐voltage cathode materials. These factors must be carefully considered when assessing electrochemical cycling performance. Notably, the increase in interface impedance and contact loss directly impacts the practical specific capacity and limits Li‐ion kinetics at high current densities.^[^ ^72^ ^]^

During reduction, we first observe the reversible formation of the LPS phase at 1.9 V, as described in Equation 8:

(8) $$Li_{4}P_{2}S_{8}+2Li^{+}+2e^{-}\to 2Li_{3}PS_{4}$$

With increasing reduction, the formation of Li_2_S and Li_n_P (0 ≤ n ≤ 3) proceeds between 1.9 to 0.6 V, under consumption of LPS, as described previously in Equation 3 and reformulated in Equation 9, assuming a stepwise progressive lithiation of P^0^ to P^1‐^, P^2‐^ and P^3‐^.

(9) $$2Li_{3}PS_{4}+\left(10+2n\right)Li^{+}+\left(10+2n\right)e^{-}\to 8Li_{2}S+2Li_{n}P$$

Unlike the polysulfide oxidation byproduct, the Li_2_S and the Li_n_P are accompanied by a sharp transition to an electrically insulating interface, and they grow on the conductive carbon (as illustrated in Figure 8a). The consequences, on one hand, are the limited electron percolation in the working electrode and an increase of the overpotential at high current density; on the other hand, electrochemically beneficial passivation that prevents further SE reduction. In addition, the LPS reduction is coupled with the working electrode volume expansion due to the overlithiated interface, which can result in cracks within the SE.^[^ ^73^ ^]^ Finally, below 0.6 V, Li_2_O is the major species dominating the interface, which we assigned to the reaction of Li_3_P with the residual oxygen inside the XPS vacuum chamber and the oxygen reduction on the VGCF surface. However, we could not exclude the formation of Li_2_O also in standard cells assembled under an Ar‐atmosphere, as traces of oxygen are always present. It is very difficult at this stage to anticipate the impact of the Li_2_O on the cycling performance, because it depends on the active materials used as an anode, in particular, Li_2_O is unstable and can easily convert to Li_2_CO_3_ or LiOH in the presence of CO/CO_2_ or H_2_O traces. Once again, it is important to consider those electro‐chemo‐mechanical instabilities when working electrodes are cycled between 2.4 and 0.0 V, using active materials like sulfur, silicon, or metallic lithium. Particularly, unlike the high voltage range, the interface in the low voltage range is more dynamic over the cycling as the Li_2_S and Li_3_P are reversibly converted back to Li_3_PS_4_ above 1.9 V (Figure 8b).

## 4 Conclusion

This study provides new insights into the electro‐chemo‐mechanical behavior of sulfide‐based solid electrolytes, particularly Li_3_PS_4,_ as a representative of the sulfide‐based SE family, under realistic battery cycling conditions. By employing *operando* X‐ray photoelectron spectroscopy (XPS) and complementary *operando* stack pressure measurements, we successfully identified and quantified the formation and evolution of oxidation and reduction byproducts, accurately measured their onset potentials, and monitored their potential‐dependency over the cycling. We determined their reversibility and elucidated their electrical properties. Furthermore, we effectively correlated the interface redox chemical changes of the Li_3_PS_4_ with the working electrode volume changes, allowing a better understanding of the mechanical instability at the solid‐solid interface.

Our methodology offers broad applicability to other SE families besides the sulfides, such as halides and oxides, capable of revealing their multiple degradation byproducts and providing a deeper understanding of the interfacial instabilities that negatively contribute to the ASSBs performance. The correlation between chemical, electrical, and mechanical responses underlines the need for advanced interface engineering strategies by optimizing, for example, material compositions or coatings, to mitigate the SE instability and unlock the full potential of ASSBs in next‐generation energy storage systems.

## 5 Experimental Section

### Composite Cathode and Cell Preparation

The cathode working electrode (WE) consisted of amorphous Li_3_PS_4_ (LPS) solid electrolyte (SE) synthesized following the procedure reported previously,^[^ ^74^, ^75^ ^]^ and conductive vapor grown carbon fiber (VGCF) provided by Resonac Holding Corporation, pre‐vacuum‐dried at 120 °C overnight. The LPS and VGCF were ball milled (Fritsch GmbH Pulverisette 7 Planetary Micro Mill) inside ZrO_2_ ball milling jars (volume 45 ml) in a weight ratio of (80:20) wt% respectively, employing ZrO_2_ balls of 5 mm diameter and a weight ratio between ZrO_2_ balls to material of 10:0.3. Ball milling was performed at a speed of 300 rpm for 5h, with a break of 10 min every 30 min. The soft ball milling conditions improved the SE‐carbon particle distribution and contact without damaging the SE. It also secured a homogeneous electron percolation in the composite WE and maximizes the amount of SE oxidation and reduction byproducts for a good signal and statistics in the XPS spectra. As a counter electrode (CE) an In‐Li alloy is used with 30 % proportional Li content. The indium foil was provided from Alfa Aesar (100 µm thickness, punched to 7 mm diameter,) and lithium foil from Goodfellow (200 µm thickness, punched to 3 mm diameter). All materials and cells assembled were manipulated and prepared inside an Ar‐filled Glovebox.

Two different custom‐made electrochemical cells were used in the study. The so‐called standard cell for the systematic electrochemical cycling evaluation of the battery materials and the *operando* XPS electrochemical cell with an open slit for the *operando* XPS measurement in ultra‐high vacuum (UHV). Both cells were validated in previous experimental work described elsewhere.^[^ ^55^, ^74^ ^]^ The corresponding cell designs can be found in Figure S14a,b, Supporting Information. Both standard and *operando* XPS cells follow the same assembly order and final stack manufacturing pressure for the preparation of pellet‐type ASSBs. At first, a polyoxymethylene (POM) cylindrical sleeve with a 7 mm inner diameter serves as an isolator and mold insert to cold‐press the stacks. For standard cells, 20 mg of LPS separator was pressed at 250 MPa, reaching an approximate thickness of 300 µm, and 5 mg of cathode composite WE was added on top with 400 MPa fabrication pressure and an approximate thickness of 100 µm (see cross‐section SEM images in Figure S14c, Supporting Information). Afterward, the indium foil is added on top of the separator as an anode together with a lithium disk on the back, corresponding to an InLi_x_ alloy of x = 0.3 with a stable redox potential of 620 mV versus Li/Li^+^.^[^ ^76^ ^]^ A thin copper foil current collector (CC) (7 mm diameter, 20 µm thickness) was placed on top of the InLi_x_ disk. The standard cell was closed with four symmetrically arranged screws and a torque key to have control over the applied stack pressure of 450 MPa during cycling.

The *operando* cell deviates so far, as the overall cell thickness was much thinner and there is no control of the final stack pressure into the open slit during the manufacturing and cycling. The SE separator was ≈200 µm thick (15 mg of LPS) and pressed together with the cathode WE composite of 40 µm thickness (2 mg loading) and an aluminum mesh as CC on top (see in‐plane‐view SEM images in Figure S14d, Supporting Information). The applied manufacturing pressure was 400 MPa for the entire ASSB stack. In the final step, the CE was mounted as previously described in the order of In/Li/Cu and pressed at 5 MPa. Afterward the cell was closed with 4 symmetrically aligned screws and tightened only by hand, as the open slit of the *operando* cell does not allow for higher pressures, with a risk of compromising the mechanical integrity of the ASSB stack. Thus, this work lacked uniaxial pressure on the ASSB stack inside the slit. Both standard and *operando* cells were kept on an open circuit potential (OCP) period to monitor the in situ formation of In‐Li alloy. Once the CE had reached a steady state after 2.5 h, the individual electrochemical cycling protocols are applied. Additionally, the *operando* cell was transferred from the Ar‐filled glovebox environment to the load lock connected to the XPS spectrometer chamber, using a custom‐made, airtight transfer chamber to avoid potential exposure to the ambient environment.

### Electrochemical Cycling Protocol

Electrochemical cycling was performed for both cells on BioLogic potentiostats, with standard cells cycling inside the Ar‐filled glovebox on BioLogic MPG‐2 multichannel and *operando* cells cycling under UHV with residual gas pressures of ≈2 × 10^−9^ mbar in the XPS chamber connected to a mono‐channel BioLogic SP‐300. Standard cell cycling performance was evaluated in cyclic voltammetry (CV) with a voltage sweep of 50 µV s^−1^. *Operando* cells were cycled in potentiostatic mode, in which predefined potential steps between 100 – 500 mV were applied, and the current was recorded over time. Once the current dropped below 1 µA (close to 2.6 µA cm^−2^), the reaction was considered to have reached a steady state, and XPS measurements were performed at the corresponding fixed cell potential. In the standard cell, during the cyclic voltammetry, constant potential electrochemical impedance spectroscopy (EIS) was measured at the end of charge at 5.0 V versus Li/Li^+^ and discharge at 2.4 V, by applying a sinusoidal AC‐voltage with an amplitude of 5 mV between 7 MHz and 100 mHz. In the EC‐lab software, an equivalent circuit consisting of a parallel combination of Resistor (R) and Constant Phase Element (CPE) for the LPS SE (R_LPS_/CPE_LPS_), the composite WE (R_LPS+VGCF_/CPE_LPS+VGCF_) and the electrode polarization (CPE_EP_) was fitted for the Nyquist plots at 5.0 V over 20 cycles. The value of R_LPS+VGCF_ was used to extract the values of resistance for the LPS:VGCF composite. In the following, all potentials measured against InLi_x_ CE are calculated against the redox potential of the Li metal electrode (−3.04 V versus standard hydrogen electrode) and reported as (V) versus Li/Li^+^ accordingly.

Complementing electrical conductivities reported in literature (see Table S7, Supporting Information), chronoamperometry in the voltage range between −50 and 50 mV (25 mV steps, 1 min current relaxation time) was conducted on pressed Li_3_PS_4_ and P_2_S_5_ pellets, between two separate stainless steel plates. The electrical conductivities were calculated using the current response, the applied voltage, the cell diameter and the individual pellet thickness.

### SEM and EDX Characterization

To evaluate and optimize the particle distribution and homogeneity of the obtained ball milled cathode composite mixture, samples were transferred from the glovebox in an airtight transfer chamber to the scanning electron microscopy (SEM) and electron dispersive X‐ray spectroscopy (EDX) measurement station. All measurements were conducted in a field emission gun equipped SEM Zeiss ULTRA 55 using the In‐lens detector of 5 kV accelerating voltage and 5 mm working distance. Parameters were kept the same for elemental mappings performed in parallel, using the Oxford Ultim Extreme EDX detector. The cross‐section shown in the SEM image of Figure S14c, Supporting Information was prepared in a Hitachi IM4000Plus Ar ion milling system utilizing a broad, low‐energy Ar^+^ ion beam and an air‐tight sample holder for the air‐protected sample transfer.

### Operando X‐Ray Photoelectron Spectroscopy Measurements and Data Processing

XPS measurements were conducted in a VG ESCALAB 220iXL spectrometer (Thermo Fisher Scientific) using a focused (spot size ≈500 µm^2^) monochromatized Al Kα radiation (1486.6 eV). A detailed methodological description of the *operando* XPS setup, including the custom‐made *operando* cell design and assembly, can be found elsewhere.^[^ ^55^ ^]^ The full battery stack was accommodated as described above in the *operando* cell, with the open lid allowing the X‐rays to penetrate the surface of the WE composite cathode, without the disturbance of a CC. Only the CC Al‐mesh pressed on top of the WE was present to ensure good electron percolation on the surface of the WE. The CC had a large enough mesh size of 0.5 mm^2^ (see Figure S14d, Supporting Information) to focus the X‐ray beam inside. The cathode WE connection was made through two copper pins connected to the metallic lid of the *operando* cell and grounded to the XPS analyzer. The body of the cell served as the connection to the In‐Li anode CE. Connections between the anode and cathode were insulated by the POM sleeve containing the full battery stack. A bias was applied between the WE and CE, with the potentiostat being set to floating mode, to not short‐circuit the system. XPS spectra were collected in a constant analyzer energy mode of pass energies at 20 eV for O1s, C1s, S2p, P2p, Al2p, Li1s, and an energy step size of 0.05 eV. No charge compensation during the measurement was necessary. The binding energies (BE) shift of all XPS core spectra plotted in the manuscript were aligned to the main peak positions acquired at OCP period for LPS, with S2p_3/2_ = 161.5 eV, P2p_3/2_ = 131.7 eV, O1s = 532.1 eV, and C1s = 284.3 eV. However, the BE shifts quantification of the different species evolving under applied bias was calculated as differences to their initial position at OCP and plotted as ΔBE to follow the evolution of their electrical properties.

Fitting of all spectra was performed in the CasaXPS software (Copyright Casa Software Ltd). For the deconvolution of different compounds, a Shirley‐type background subtraction was applied, and the peaks fitted with a sum of Gaussian (70%) and Lorentzian (30%) line shapes in the Marquardt fit method under application of a relative sensitivity factor of 1 and a root‐mean‐square figure of merit. Residual standard deviation (RSD) was close to unity for S2p, O1s, C1s, Al2p, and Li1s. For P2p, the RSD can reach up to 2 due to the worsened signal‐to‐noise ratio. The spin‐orbit split (ΔE) and branching ratio (β) of the S2p_3/2+1/2_ and P2p_3/2+1/2_ were fixed to ΔE_S2p_ = 1.2 eV, β_S2p_ = 0.5, ΔE_P2p_ = 0.87 eV, and β_P2p_ = 0.5. For Al2p_3/2+1/2_ of metallic aluminum and aluminum oxide, the ΔE_Al2p_ = 0.44 eV and β_Al2p_ = 0.5 were applied. Contributions of the S2p_1/2_ and P2p_1/2_ spin‐orbits were subtracted by application of the method developed by *Himpsel* et al.^[^ ^77^ ^]^ for the Si2p deconvolution using Thermo Avantage software (V 4.87), as illustrated in Figure S15a, Supporting Information for the OCP composite WE and its core level spectra of S2p and P2p. Furthermore, the Al2s plasmon loss peak located at 133 eV overlaps with the P2p peak from the SE and prevents the accurate analysis of the changes taking place in the P2p core level, as shown in Figure S15b, Supporting Information. Therefore, the background signal of the Al2s and its corresponding plasmon loss peaks evolving from the Al‐mesh was subtracted in Thermo Avantage software from each of the P2p core level spectra (see Figure S15b, Supporting Information). The binding energy (BE) and the full‐width‐half‐maximum (FWHM) of the fitted S2p_3/2_ and P2p_3/2_ components collected at the different applied potentials are reported in Table S1, Supporting Information (high voltage range) and Tables S4 and S5, Supporting Information (low voltage range). As all fitted compounds experienced in the low voltage range a certain BE shift dependent on the applied voltage, their voltage‐dependent shifts were corrected respectively to their BE position at OCP and their exact BE positions are listed in Table S6, Supporting Information. The peak area ratio of the fitted compounds in the S2p_3/2_ and P2p_3/2_ spin‐orbits was translated to atomic % by dividing the compound's peak area by the calculated sensitivity factors (see Equation S1, Supporting Information) of S2p_3/2_ of S─P, P‐S‐S‐P, Li_2_S and P2p_3/2_ of Li_n_P and are listed for both high and low voltage range components in Table S2, Supporting Information.

### Operando Stack Pressure Monitoring

The cell pressure evolution during cycling was measured using CompreFrame (rhd instruments GmbH & Co. KG), in which the force sensor has a range from 0 to 10 kN (8.84 MPa) with the linearity error ≤ ± 0.1 %. The ASSB stack was assembled in a polyetheretherketone (PEEK) sleeve of 12 mm inner diameter using a uniaxial press inside the Ar‐filled glovebox. 70 mg of LPS was prepared as a separator and 15.2 mg of LPS:VGCF, 80:20 wt% composite mixture as WE. Then, 22.5 mg of Li_4_Ti_5_O_12_ (LTO) (Süd‐Chemie):Li_6_PS_5_Cl (LPSCl) (NEI Corporation) mixture was prepared as a CE (with mass ratio LTO:LPSCl, 70:30 wt%, ball milled for 30 min). All powders for the LPS separator, the WE and CE composites were compressed at 380 MPa for 1 min to form a dense pellet. The initial applied stack pressure was 80 MPa by CompreFrame. LTO as CE delivered a stable OCP at 1.5 V versus Li/Li^+^. Before cycling, the cell rested for 3 h to minimize the effect of the cell pressure decay related to the cell mechanical relaxation (see Figure S12, Supporting Information). CV measurements were then performed at a scan rate of 50 µV s^−1^ at room temperature. Two voltage ranges were selected comparable to the high and low voltage cycling protocol of the *operando* XPS. Starting with oxidation, two cycles in the high voltage range (2.4–5.0 V) are collected, followed by two cycles in the low voltage range (2.4–1.0 V), and finished with a final charge from 2.4 to 5.0 V. The real‐time pressure monitoring was realized by the control software of CompreFrame.

## Conflict of Interest

The authors declare no conflict of interest.

## Supporting information



Supporting Information

## Data Availability

The data that support the findings of this study are openly available in Zenodo at https://doi.org/10.5281/zenodo.15606971, and upon reasonable request.
