# Supplementary material for: Electrochemical and Mechanical Evolution of Sulfide‐Based Solid Electrolytes: Insights from Operando XPS and Cell Pressure Measurements
Source: Small. 2025 Oct 3;21(46):e08796. doi: 10.1002/smll.202508796 (PMC12632433; doi:10.1002/smll.202508796)
Supplement: Supplementary file 1 — Supporting Information [file SMLL-21-e08796-s001.docx]

Supporting Information

Electrochemical and Mechanical Evolution of Sulfide-Based Solid Electrolytes: Insights from Operando XPS and Cell Pressure Measurements

Valerie Siller,* Linfeng Xu, Laurent Castro, Aurélie Guéguen and Mario El Kazzi*


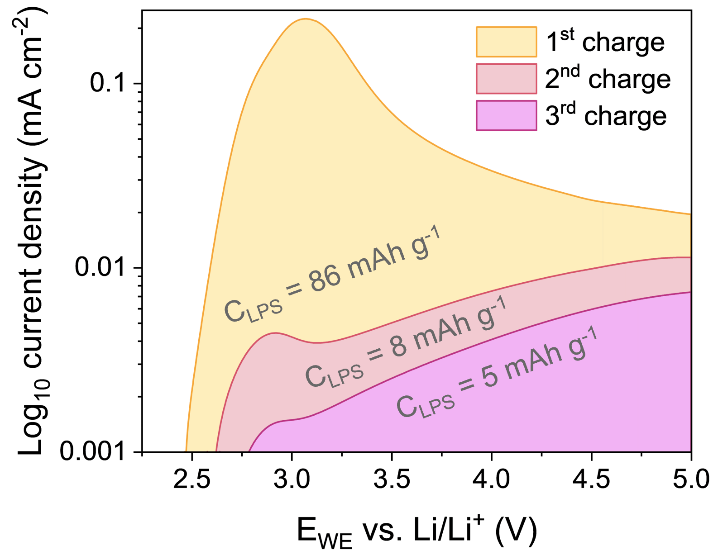


**Figure S1.** Logarithmic current density upon charge for first, second, and third charge in the high voltage range and the corresponding calculated specific capacities normalized to the 80 wt% LPS mass in the composite WE (LPS:VGCF, 80:20 wt%).


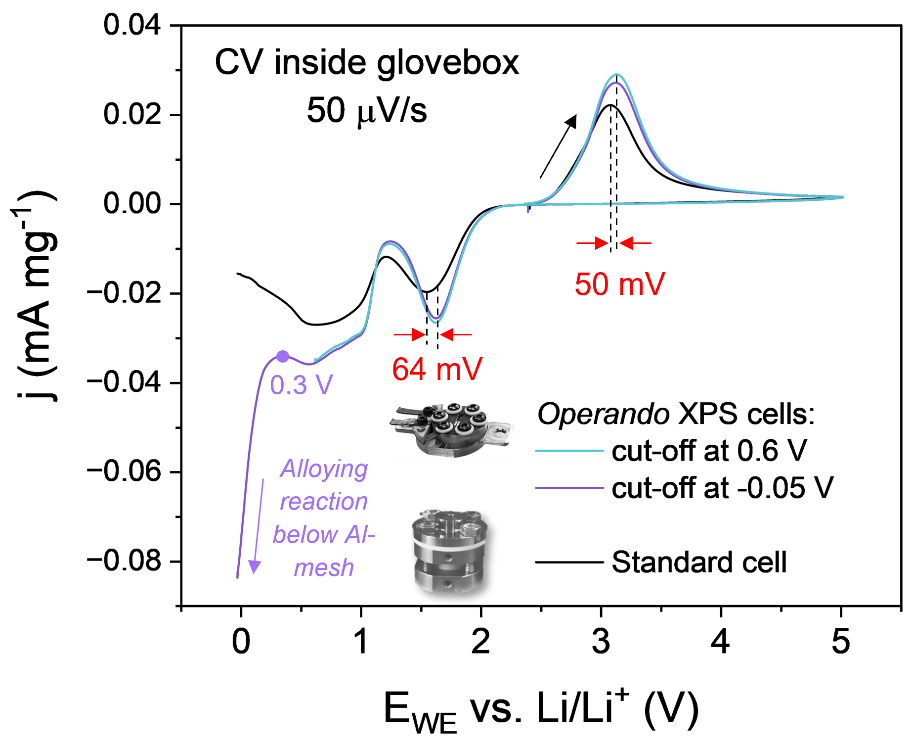


**Figure S2.** Cyclic voltammetry curves of the WE (LPS:VGCF, 80:20 wt%) cycled with 50 µV s^-1^ sweep rate in the high and low voltage range of the standard electrochemical cell (black) and the operando XPS cell (blue and purple) inside the glovebox. The overpotentials between the standard and the operando cell are marked in red. The operando XPS cell was cycled to two different cut-off potentials in the low voltage range, to evaluate the influence of the alloying reaction of lithium with the Al-CC mesh.


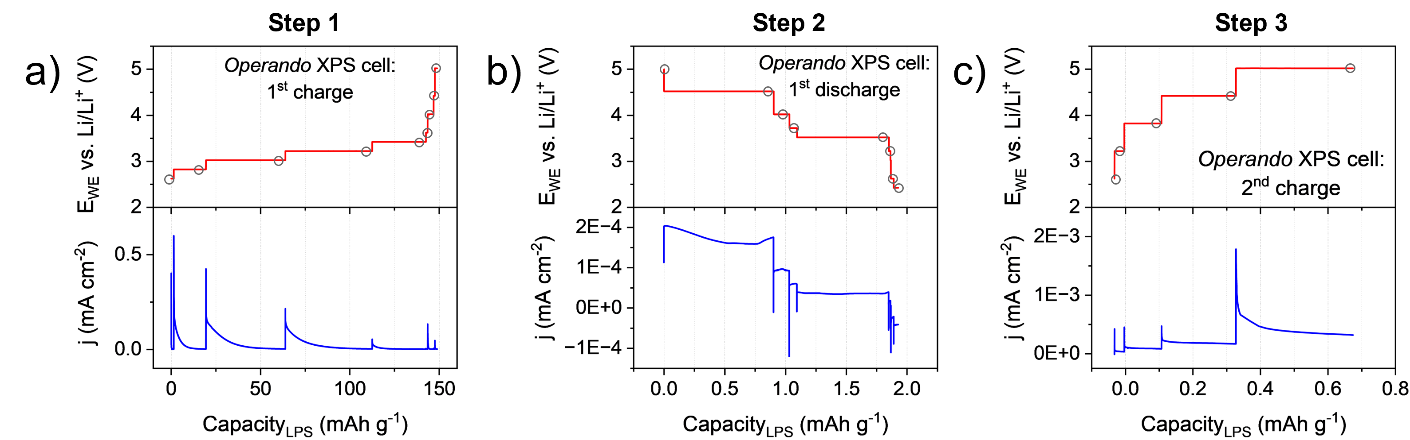


**Figure S3.** Operando XPS cycling protocol performed on the WE (LPS:VGCF, 80:20 wt%). Voltage (red) and current density (dark blue) profiles over the specific capacity normalized to 80 wt% LPS of (a) step 1, (b) step 2, and (c) step 3, with the corresponding potential steps for operando XPS measurements marked as circles (grey).


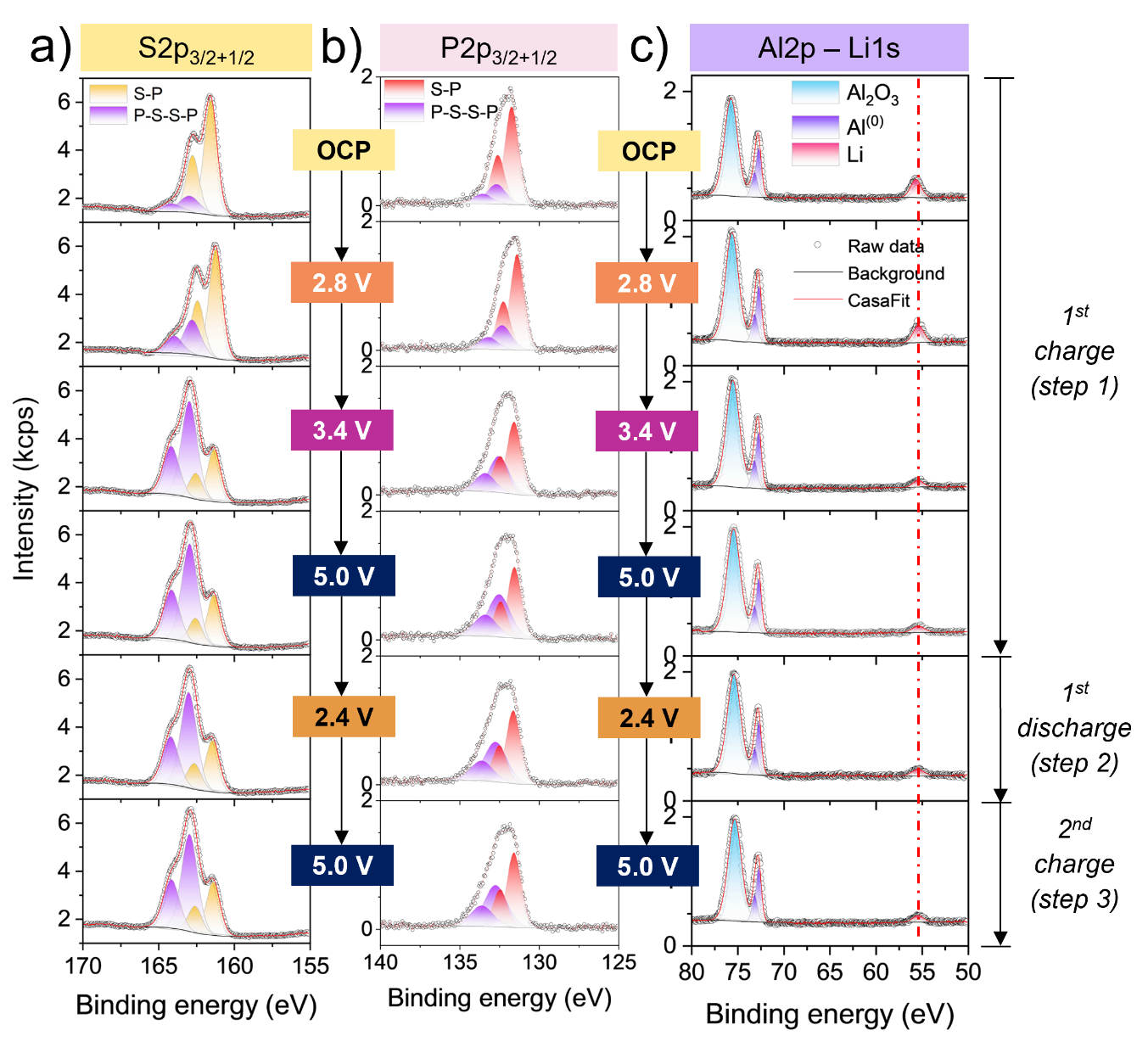


**Figure S4.** Core level spectra acquired on the WE (LPS:VGCF, 80:20 wt%) at various potentials in the high voltage range for (a) S2p, (b) P2p, and (c) Al2p-Li1s with the fitted compounds, raw data, background, and envelope of the fitting. The red dash-dotted line in (c) serves as a guide for the eye for the minor voltage-dependent binding energy position shift in the Li1s peak position. Voltage-dependent shifts in binding energies are not corrected.

**Table S1.** A list of the parameters used for the fitting in CasaXPS of the S2p_3/2_, P2p_3/2_ and Li1s corresponding compounds acquired on the WE (LPS:VGCF, 80:20 wt%) cycled in the high voltage range, with reference to the as-synthesized Li_3_PS_4_ and the pristine composite WE pellet. All values in red have been fixed to a maximum or minimum in binding energy position or full width half maximum (FWHM), while all values in black have not been fixed.

| **E_WE_ vs. Li/Li^+^ (V)** | **Binding energy position (eV)** | | | | | **FWHM (eV)** | | | | |
| --- | --- | --- | --- | --- | --- | --- | --- | --- | --- | --- |
|  | S2p 3/2 | | P2p 3/2 | | Li1s | S2p 3/2 | | P2p 3/2 | | Li1s |
|  | S-P | P-S-S-P | S-P | P-S-S-P | Li | S-P | P-S-S-P | S-P | P-S-S-P | Li |
| Reference Li_3_PS_4_ | 161.3 | 162.3 | 131.6 | 132.5 | 55.2 | 1.0 | 1.3 | 1.0 | 0.9 | 1.4 |
| LPS:VGCF WE pellet | 161.6 | 163.2 | 131.9 | 132.7 | 55.6 | 1.0 | 1.6 | 1.0 | 1.4 | 1.3 |
| 2.4 | 161.5 | 162.9 | 131.7 | 132.6 | 55.6 | 1.0 | 1.6 | 1.0 | 1.4 | 1.3 |
| 2.6 | 161.4 | 162.9 | 131.6 | 132.5 | 55.4 | 1.0 | 1.5 | 1.0 | 1.3 | 1.4 |
| 2.8 | 161.2 | 162.7 | 131.3 | 132.3 | 55.3 | 1.0 | 1.3 | 1.0 | 1.4 | 1.3 |
| 3 | 161.1 | 162.7 | 131.3 | 132.3 | 55.2 | 1.0 | 1.3 | 1.0 | 1.4 | 1.5 |
| 3.2 | 161.1 | 162.8 | 131.4 | 132.3 | 55.2 | 1.0 | 1.3 | 1.1 | 1.6 | 1.6 |
| 3.4 | 161.3 | 162.9 | 131.6 | 132.5 | 55.3 | 1.0 | 1.2 | 1.1 | 1.7 | 1.3 |
| 3.6 | 161.3 | 162.9 | 131.6 | 132.6 | 55.4 | 1.0 | 1.3 | 1.1 | 1.7 | 1.5 |
| 4 | 161.2 | 162.8 | 131.5 | 132.5 | 55.3 | 1.0 | 1.2 | 1.1 | 1.7 | 1.7 |
| 4.4 | 161.3 | 162.9 | 131.5 | 132.5 | 55.3 | 1.0 | 1.3 | 1.1 | 1.7 | 1.4 |
| 5 | 161.3 | 162.9 | 131.5 | 132.5 | 55.3 | 1.0 | 1.2 | 1.0 | 1.7 | 1.6 |
| 4.5 | 161.3 | 162.9 | 131.5 | 132.5 | 55.3 | 1.1 | 1.2 | 1.1 | 1.7 | 1.5 |
| 4 | 161.4 | 162.9 | 131.6 | 132.6 | 55.4 | 1.1 | 1.2 | 1.1 | 1.7 | 1.5 |
| 3.7 | 161.3 | 162.9 | 131.5 | 132.6 | 55.3 | 1.1 | 1.2 | 1.1 | 1.7 | 1.7 |
| 3.5 | 161.3 | 162.9 | 131.6 | 132.7 | 55.3 | 1.1 | 1.3 | 1.1 | 1.7 | 1.6 |
| 3.2 | 161.3 | 162.9 | 131.6 | 132.7 | 55.3 | 1.1 | 1.3 | 1.1 | 1.7 | 1.4 |
| 3 | 161.4 | 162.9 | 131.6 | 132.7 | 55.4 | 1.1 | 1.3 | 1.1 | 1.7 | 1.5 |
| 2.6 | 161.4 | 162.9 | 131.6 | 132.7 | 55.4 | 1.1 | 1.3 | 1.1 | 1.7 | 1.7 |
| 2.4 | 161.4 | 162.9 | 131.6 | 132.7 | 55.3 | 1.1 | 1.3 | 1.1 | 1.7 | 1.6 |
| 2.6 | 161.4 | 162.9 | 131.6 | 132.7 | 55.4 | 1.1 | 1.3 | 1.1 | 1.7 | 1.5 |
| 3.2 | 161.4 | 162.9 | 131.6 | 132.7 | 55.4 | 1.1 | 1.3 | 1.1 | 1.7 | 1.9 |
| 3.8 | 161.4 | 162.9 | 131.6 | 132.7 | 55.4 | 1.1 | 1.3 | 1.1 | 1.7 | 1.6 |
| 4.4 | 161.3 | 162.9 | 131.6 | 132.7 | 55.3 | 1.1 | 1.3 | 1.1 | 1.7 | 1.8 |
| 5 | 161.3 | 162.9 | 131.5 | 132.7 | 55.3 | 1.1 | 1.3 | 1.1 | 1.7 | 1.4 |


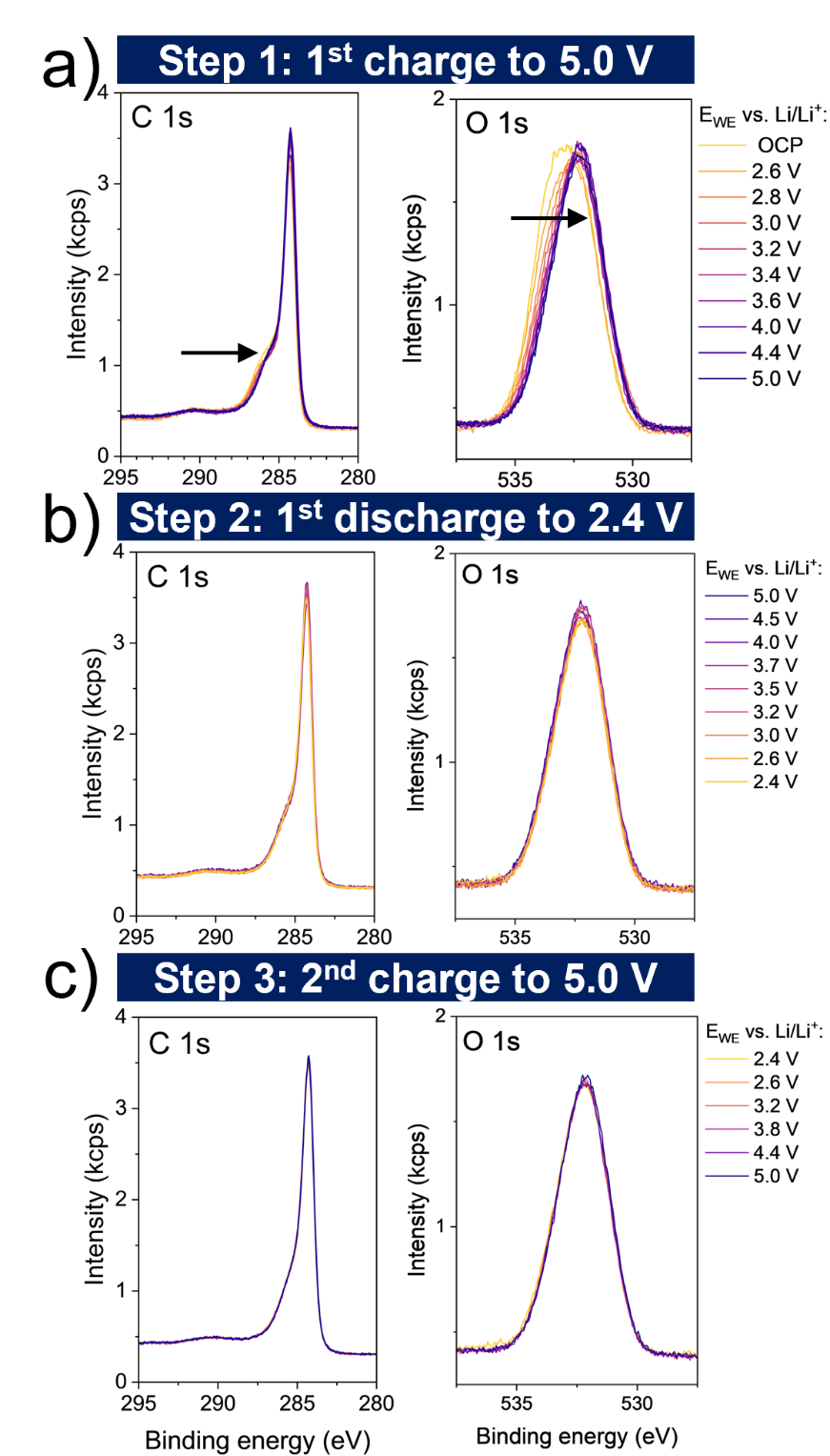


**Figure S5.** C1s and O1s core level spectra acquired on the WE (LPS:VGCF, 80:20 wt%) in the high voltage range at different potentials for (a) step 1, (b) step 2 and (c) step 3. All spectra are shown as raw data, without any voltage-dependent BE shift correction.


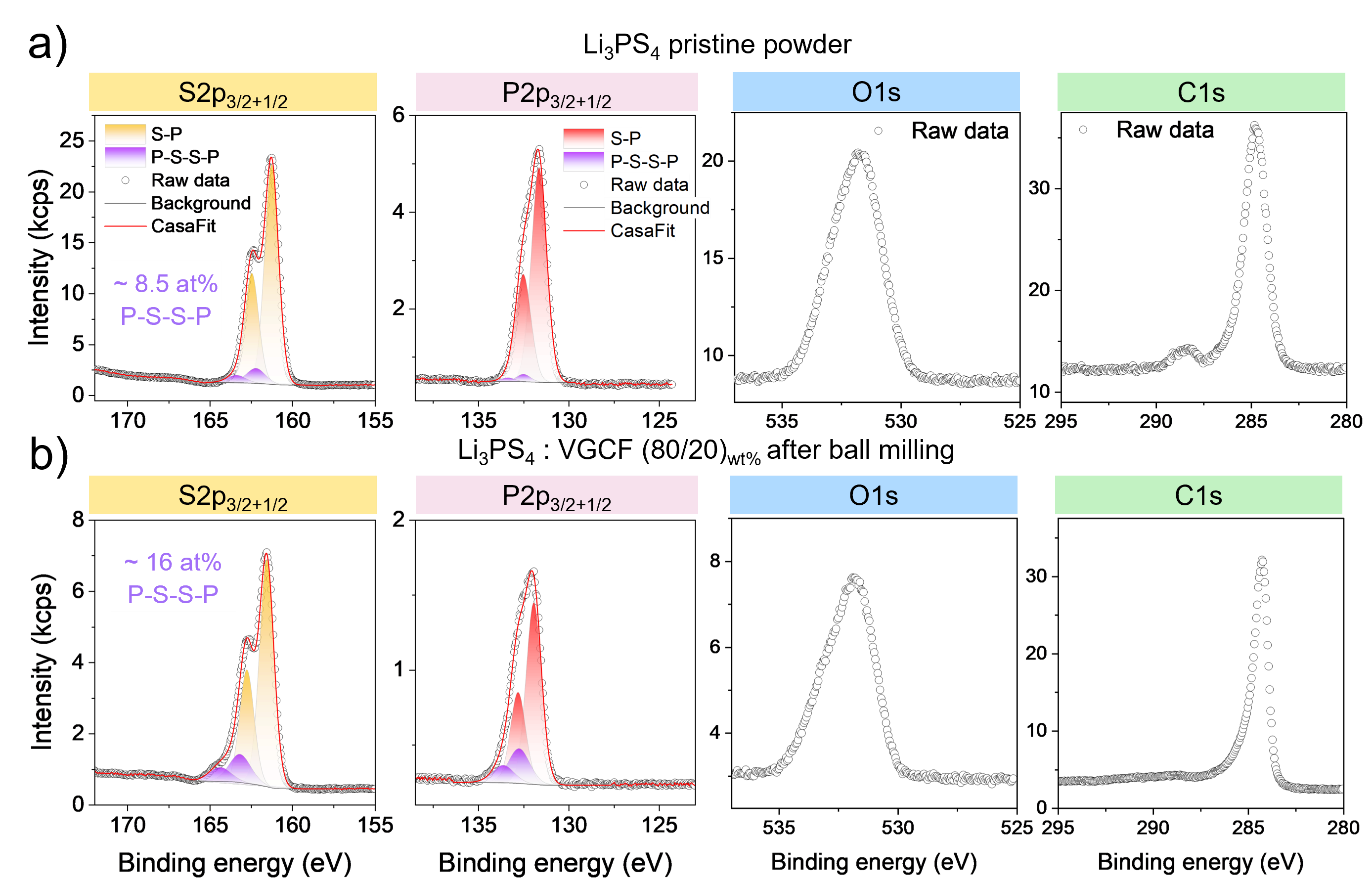


**Figure S6.** Comparison of S2p, P2p, O1s, and C1s core level spectra for (a) Li_3_PS_4_ pristine powder and (b) the ball milled composite WE of LPS:VGCF (80:20 wt%). The calculated atomic % of the oxidized polysulfides (P-S-S-P) is shown in purple.

**Table S2.** Calculated atomic % (at%) for all compounds at each potential in the high voltage and low voltage range. Calculations are based on the fitted area below the compounds S2p_3/2_ and P2p_3/2_ peaks and divided by the corresponding relative sensitivity factor described in Equation S1 for S2p and P2p of LPS, with subsequent normalization to 100 %.

| **High voltage range** | | | **Low voltage range** | | | | |
| --- | --- | --- | --- | --- | --- | --- | --- |
| **E_WE_ vs. Li/Li^+^ (V)** | **S-P**  **(at%)** | **P-S-S-P**  **(at%)** | **E_WE_ vs. Li/Li^+^ (V)** | **S-P**  **(at%)** | **P-S-S-P**  **(at%)** | **Li_2_S**  **(at%)** | **Li_n_P**  **(at%)** |
| Reference Li_3_PS_4_ | 91 | 9 | 5.0 | 32 | 68 | 0 | 0 |
| LPS:VGCF WE pellet | 84 | 16 | 4.2 | 31 | 69 | 0 | 0 |
| 2.4 | 82 | 18 | 3.4 | 31 | 69 | 0 | 0 |
| 2.6 | 83 | 17 | 2.4 | 31 | 69 | 0 | 0 |
| 2.8 | 71 | 29 | 2.0 | 31 | 69 | 0 | 0 |
| 3.0 | 49 | 51 | 1.9 | 69 | 29 | 2 | 0 |
| 3.2 | 35 | 65 | 1.8 | 71 | 25 | 3 | 1 |
| 3.4 | 31 | 69 | 1.7 | 73 | 18 | 7 | 3 |
| 3.6 | 31 | 69 | 1.5 | 69 | 17 | 10 | 4 |
| 4.0 | 30 | 70 | 1.2 | 48 | 16 | 26 | 10 |
| 4.4 | 30 | 70 | 1.0 | 19 | 8 | 52 | 21 |
| 5.0 | 30 | 70 | 0.8 | 12 | 5 | 57 | 25 |
| 4.5 | 31 | 69 | 0.6 | 12 | 4 | 61 | 22 |
| 4.0 | 31 | 69 | 1.5 | 10 | 5 | 61 | 24 |
| 3.7 | 31 | 69 | 1.9 | 15 | 6 | 56 | 22 |
| 3.5 | 31 | 69 | 2.4 | 46 | 17 | 24 | 13 |
| 3.2 | 31 | 69 | 2.6 | 55 | 35 | 7 | 3 |
| 3.0 | 31 | 69 | 0.6 | 17 | 6 | 59 | 18 |
| 2.6 | 31 | 69 | 0.3 | 11 | 3 | 63 | 23 |
| 2.4 | 32 | 68 | 0.1 | 13 | 5 | 56 | 26 |
| 2.6 | 31 | 69 | -0.05 | 11 | 4 | 52 | 33 |
| 3.2 | 32 | 68 |  |  |  |  |  |
| 3.8 | 32 | 68 |  |  |  |  |  |
| 4.4 | 32 | 68 |  |  |  |  |  |
| 5.0 | 32 | 68 |  |  |  |  |  |

***Formula for calculating atomic % (at%) of component A:***

$A(at\%)=\left[ \left( \frac{I_{A}}{s_{A}} \right)/\sum\left( \frac{I_{i}}{s_{i}} \right) \right]*100 \%$ (**Equation S1a**)

$s_{A}=\sigma_{A}T_{A}\lambda_{A}$ = relative sensitivity factor (R.S.F.) (**Equation S1b**)

With:

σ = cross-section derived from Avantage Scofield from Al K_α_ = 1486.6 eV for each element

T = Transmission and instrument function = TXFN from Avantage for each element

λ = inelastic mean free path = IMFP calculated from NIST Electron Inelastic-Mean-Free-Path Database Version 1.2^[1]^

**For Sulfur S2p_3/2_ compounds of S-P, P-S-S-P and Li_2_S:** $\sigma_{S}=1.11$; $T_{S}=3370.43$; $\lambda_{S}=3.168 nm$

**For Phosphorus P2p_3/2_ compounds of Li_n_P:** $\sigma_{P}=0.789$; $T_{P}=3348.82$; $\lambda_{P}=3.225 nm$

**Table S3.** Binding energy positions of S2p core level compounds evolving upon oxidation of sulfide solid electrolyte composites.

| **Reference S2p BE position** | **Material: Composite (composition, mixing)** | **BE S^0^**  **(eV)** | **BE P-S-S-P (eV)** | **BE PS_4_^3-^**  **(eV)** | **BE Li_2_S**  **(eV)** | **ΔBE S^0^ - PS_4_^3-^ (eV)** | **ΔBE P-S-S-P - PS_4_^3-^ (eV)** | **ΔBE Li_2_S - PS_4_^3-^ (eV)** | **ΔBE Li_2_S - S^0^ (eV)** | **ΔBE Li_2_S - P-S-S-P (eV)** |
| --- | --- | --- | --- | --- | --- | --- | --- | --- | --- | --- |
| Auvergniot et al.^[2]^ | LiCoO_2_ / Li_6_PS_5_Cl / VGCF (38/57/5, HM) | 164.4 | 163.5 | 161.7 | 160.5 | **2.7** | **1.8** | **-1.2** | **-3.9** | **-3.0** |
| Dewald et al.^[3]^ | Li_2_S:P_2_S_5_ / C65  (90/10, HM) | 163.4 | 162.9 | 161.5 | 160 | **1.9** | **1.4** | **-1.5** | **-3.4** | **-2.9** |
| Tan et al.^[4]^ | Li_6_PS_5_Cl / C  (70/30, BM) | 163.2 | **--** | 161.5 | 160.3 | **1.7** | **--** | **-1.2** | **-2.9** | **--** |
| Wang et al.^[5]^ | Li_6_PS_5_Cl_0.5_Br_0.5_/MWCNT (70/30, BM) | 163.6 | 162.9 | 161.7 | 160.2 | **1.9** | **1.2** | **-1.5** | **-3.4** | **-3.4** |
| Nagai et al.^[6]^ | S/CF/Li_3_PS_4_ (35.9/20.5/43.6, HM) | 164 | 163.5 | 162 | 160.7 | **2** | **1.5** | **-1.3** | **-3.3** | **-2.8** |
| Wang et al.^[7]^ | β-Li_3_PS_4_ / C65  (75/25, HM) | 164 | 163 | 161.7 | -- | **2.3** | **1.3** | **--** | **--** | **--** |
| Walther et al.^[8]^ | NCM_622_/β-Li_3_PS_4_/VGCF  (67.9/29.1/3.0, HM) | 164 | 163 | 161.7 | -- | **2.3** | **1.3** | **--** | **--** | **--** |
| Koerver et al.^[9]^ | NCM_811_ / β-Li_3_PS_4_  (70/30, HM) | 163.5 | 162.7 | 161.4 | 159.8 | **2.1** | **1.3** | **-1.6** | **-3.7** | **-2.9** |
| Nguyen et al.^[10]^ | Na_4_(B_10_H_10_)(B_12_H_12_)/C/S  (3/1/2, BM) | 163.6 (elemental sulfur) | 162.1  (Na_2_S_x_; 2 ≤ x ≤ 8) | **--** | 160.6  (Na_2_S) | **--** | **--** | **--** | **-3**  (Na_2_S) | **-1.5**  (Na2Sx; 2 ≤ x ≤ 8) |
| This work | Li_3_PS_4_ / VGCF  (80/20, BM) | **--** | 162.9 | 161.5 | 160 | **--** | **1.4** | **-1.5** | **--** | **-2.9** |


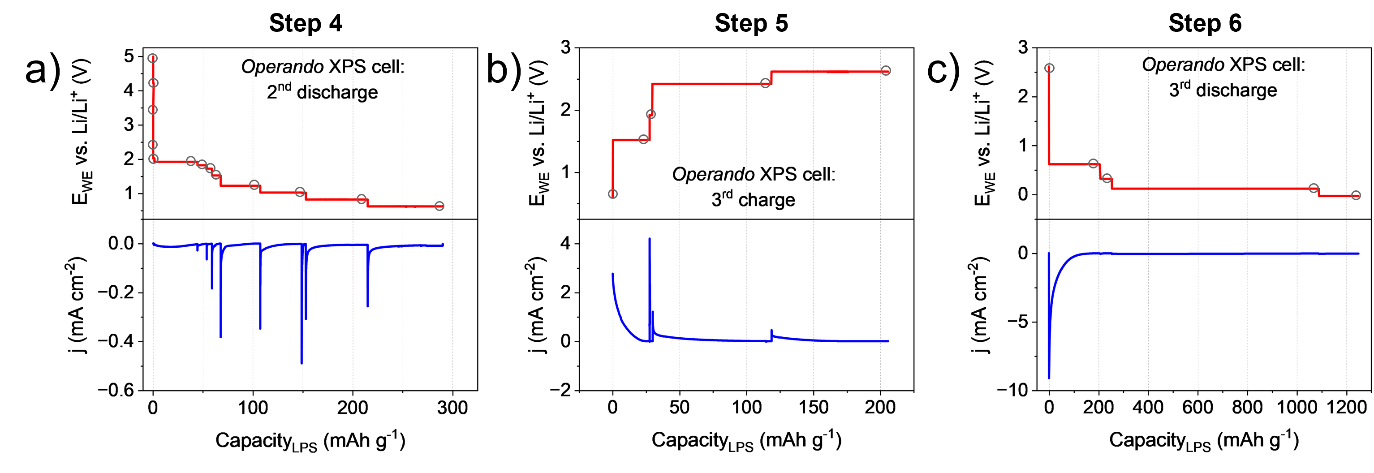


**Figure S7.** Operando XPS cycling protocol performed on the WE (LPS:VGCF, 80:20 wt%). Voltage (red) and current density (dark blue) profiles over the specific capacity normalized to 80 wt% LPS. (a) step 4, (b) step 5 and (c) step 6, with the corresponding potential steps for operando XPS measurements marked as circles (grey).


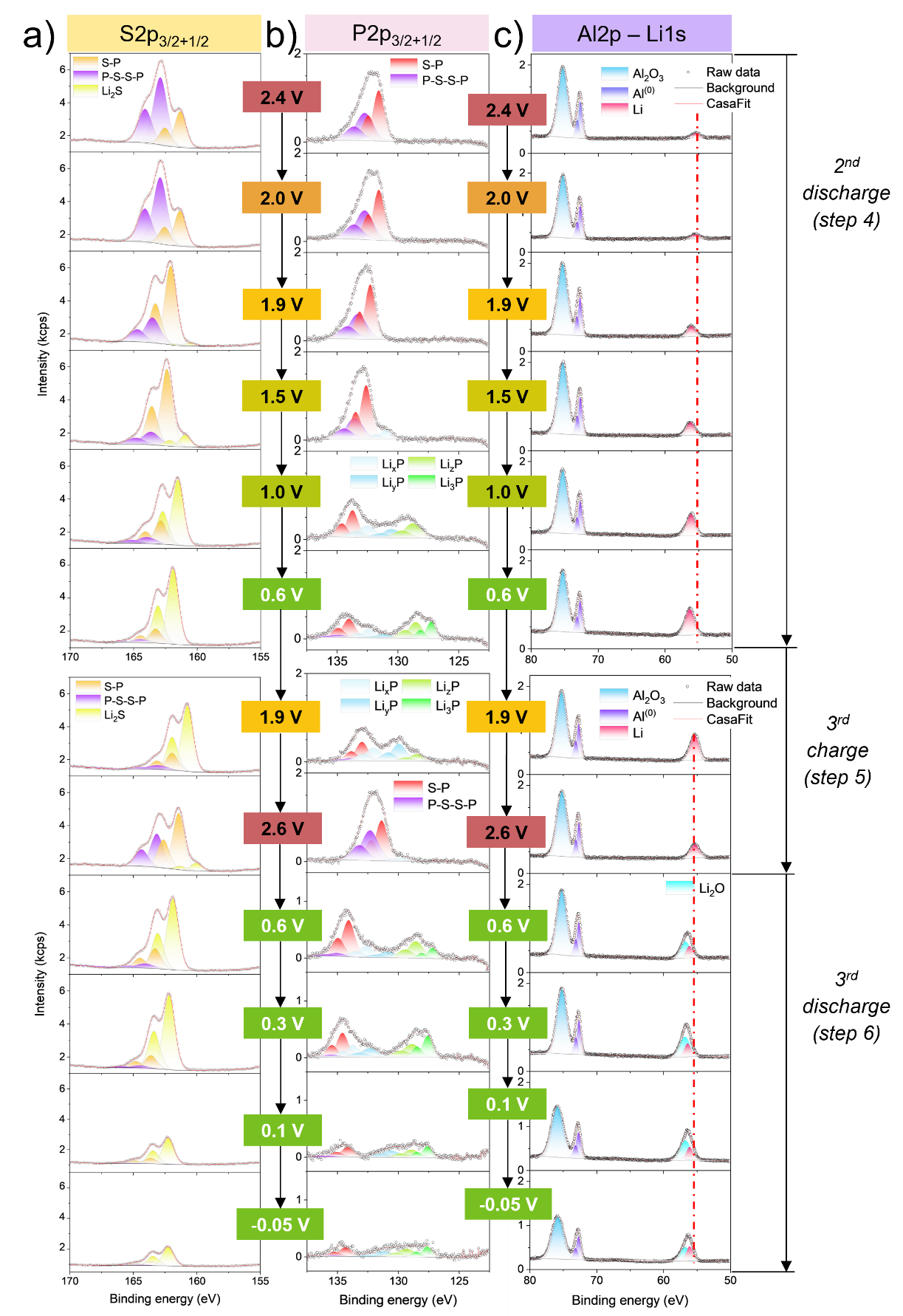


**Figure S8.** Core level spectra acquired on the WE (LPS:VGCF, 80:20 wt%) at various potentials in the low voltage range for (a) S2p, (b) P2p, and (c) Al2p-Li1s with the fitted compounds, raw data, background, and envelope of the fitting. The red dash-dotted line in (c) serves as a guide for the eye for the minor voltage-dependent binding energy position shift in the Li1s peak position. Voltage-dependent shifts in binding energies are not corrected.

**Table S4.** A list of the binding energy positions used for the fitting in CasaXPS of the S2p_3/2_, P2p_3/2_, Li1s and O1s corresponding compounds acquired on the WE (LPS:VGCF, 80:20 wt%) cycled in the low voltage range, with reference to the as-synthesized Li_3_PS_4_. All values in red have been fixed to a maximum or minimum in binding energy position, while all values in black have not been fixed.

|  | **Binding energy position (eV)** | | | | | | | | | | | | |
| --- | --- | --- | --- | --- | --- | --- | --- | --- | --- | --- | --- | --- | --- |
| **E_WE_ vs. Li/Li^+^** | S2p 3/2 | | | P2p 3/2 | | | | | | Li1s | | O1s | |
| **(V)** | S-P | P-S-S-P | Li_2_S | S-P | P-S-S-P | Li_x_P | Li_y_P | Li_z_P | Li_3_P | Li | Li_2_O | Li_2_O |  |
| Reference Li_3_PS_4_ | 161.3 | 162.3 |  | 131.6 | 132.5 |  |  |  |  | 55.2 |  |  |  |
| 5 | 161.3 | 162.9 |  | 131.5 | 132.7 |  |  |  |  | 55.3 |  |  |  |
| 4.2 | 161.2 | 162.8 |  | 131.5 | 132.6 |  |  |  |  | 55.2 |  |  |  |
| 3.4 | 161.3 | 162.9 |  | 131.5 | 132.7 |  |  |  |  | 55.3 |  |  |  |
| 2.4 | 161.3 | 162.9 |  | 131.5 | 132.7 |  |  |  |  | 55.3 |  |  |  |
| 2 | 161.3 | 162.9 |  | 131.5 | 132.7 |  |  |  |  | 55.3 |  |  |  |
| 1.9 | 162.1 | 163.5 | 160.5 | 132.2 | 133.2 |  |  |  |  | 56.0 |  |  |  |
| 1.8 | 162.1 | 163.5 | 160.6 | 132.3 | 133.4 | 130.8 |  |  |  | 56.0 |  |  |  |
| 1.7 | 162.3 | 163.6 | 160.8 | 132.5 | 133.4 | 130.7 |  |  |  | 56.1 |  |  |  |
| 1.5 | 162.4 | 163.6 | 160.9 | 132.6 | 133.5 | 130.9 |  |  |  | 56.1 |  |  |  |
| 1.2 | 162.7 | 163.7 | 161.2 | 133.0 | 133.9 | 131.1 | 129.4 |  |  | 56.1 |  |  |  |
| 1 | 162.8 | 163.9 | 161.5 | 133.7 | 134.7 | 132.5 | 130.5 | 128.7 |  | 56.0 |  |  |  |
| 0.8 | 163.0 | 164.1 | 161.7 | 134.0 | 134.9 | 132.6 | 130.9 | 128.3 | 126.9 | 56.0 |  |  |  |
| 0.6 | 163.2 | 164.4 | 161.9 | 134.0 | 135.0 | 132.4 | 131.1 | 128.5 | 127.2 | 56.2 |  |  |  |
| 1.5 | 162.4 | 163.6 | 161.1 | 133.4 | 134.3 | 132.3 | 130.0 | 128.7 | 127.7 | 55.6 |  |  |  |
| 1.9 | 161.9 | 163.1 | 160.7 | 133.0 | 133.9 | 132.0 | 129.9 | 128.5 |  | 55.3 |  |  |  |
| 2.4 | 161.7 | 163.3 | 160.3 | 132.1 | 133.0 | 131.3 | 129.7 |  |  | 55.2 |  |  |  |
| 2.6 | 161.4 | 163.1 | 160.0 | 131.3 | 132.3 | 129.6 |  |  |  | 55.2 |  |  |  |
| 0.6 | 163.3 | 164.1 | 161.9 | 134.1 | 135.0 | 132.6 | 130.9 | 128.5 | 127.1 | 56.1 | 56.7 | 529.7 |  |
| 0.3 | 163.6 | 164.5 | 162.2 | 134.6 | 135.5 | 133.7 | 132.3 | 128.8 | 127.6 | 56.3 | 56.7 | 530.6 |  |
| 0.1 | 163.6 | 164.7 | 162.2 | 134.2 | 135.1 |  | 130.6 | 128.9 | 127.6 | 56.0 | 56.6 | 530.9 |  |
| -0.05 | 163.6 | 164.6 | 162.2 | 134.3 | 135.3 |  | 131.2 | 129.3 | 127.6 | 56.0 | 56.7 | 530.9 |  |

**Table S5.** A list of the full width at half maximum (FWHM) used for the fitting in CasaXPS of the S2p_3/2_, P2p_3/2_, Li1s and O1s corresponding compounds acquired on the WE (LPS:VGCF, 80:20 wt%) cycled in the low voltage range, with reference to the as-synthesized Li_3_PS_4_. All values in red have been fixed to a maximum or minimum in FWHM, while all values in black have not been fixed.

|  | **FWHM (eV)** | | | | | | | | | | | | |
| --- | --- | --- | --- | --- | --- | --- | --- | --- | --- | --- | --- | --- | --- |
| **E_WE_ vs. Li/Li^+^** | S2p 3/2 | | | P2p 3/2 | | | | | | Li1s | | O1s | |
| **(V)** | S-P | P-S-S-P | Li_2_S | S-P | P-S-S-P | Li_x_P | Li_y_P | Li_z_P | Li_3_P | Li | Li_2_O | Li_2_O |  |
| Reference Li_3_PS_4_ | 1.0 | 1.3 |  | 1.0 | 0.9 |  |  |  |  | 1.4 |  |  |  |
| 5 | 1.1 | 1.3 |  | 1.1 | 1.7 |  |  |  |  | 1.4 |  |  |  |
| 4.2 | 1.1 | 1.3 |  | 1.1 | 1.7 |  |  |  |  | 1.5 |  |  |  |
| 3.4 | 1.1 | 1.3 |  | 1.1 | 1.7 |  |  |  |  | 1.6 |  |  |  |
| 2.4 | 1.1 | 1.3 |  | 1.1 | 1.7 |  |  |  |  | 1.5 |  |  |  |
| 2 | 1.1 | 1.3 |  | 1.1 | 1.7 |  |  |  |  | 1.9 |  |  |  |
| 1.9 | 1.1 | 1.4 | 1.0 | 1.0 | 1.7 |  |  |  |  | 1.5 |  |  |  |
| 1.8 | 1.1 | 1.5 | 1.0 | 1.0 | 1.7 | 1.0 |  |  |  | 1.5 |  |  |  |
| 1.7 | 1.1 | 1.4 | 1.0 | 1.1 | 1.7 | 1.0 |  |  |  | 1.6 |  |  |  |
| 1.5 | 1.1 | 1.6 | 1.0 | 1.1 | 1.7 | 1.1 |  |  |  | 1.5 |  |  |  |
| 1.2 | 1.1 | 1.8 | 1.1 | 1.1 | 1.6 | 1.3 | 1.7 |  |  | 1.7 |  |  |  |
| 1 | 1.1 | 1.7 | 1.1 | 1.1 | 1.6 | 1.4 | 1.7 | 1.6 |  | 1.5 |  |  |  |
| 0.8 | 1.1 | 1.5 | 1.0 | 1.1 | 1.6 | 1.3 | 1.7 | 1.6 | 0.9 | 1.5 |  | 1.2 |  |
| 0.6 | 1.1 | 1.5 | 1.0 | 1.2 | 1.6 | 1.1 | 1.7 | 1.4 | 0.9 | 1.6 |  | 1.2 |  |
| 1.5 | 1.1 | 1.7 | 1.1 | 1.1 | 1.7 | 1.5 | 1.5 | 1.3 | 1.0 | 1.5 |  | 1.2 |  |
| 1.9 | 1.1 | 1.8 | 1.0 | 1.2 | 1.6 | 1.5 | 1.3 | 1.3 |  | 1.5 |  | 1.2 |  |
| 2.4 | 1.1 | 1.2 | 1.0 | 1.2 | 1.6 | 1.0 | 1.6 |  |  | 1.8 |  |  |  |
| 2.6 | 1.1 | 1.2 | 1.1 | 1.2 | 1.6 | 1.5 |  |  |  | 1.6 |  |  |  |
| 0.6 | 1.1 | 1.7 | 1.1 | 1.2 | 1.6 | 1.3 | 1.7 | 1.5 | 0.9 | 1.2 | 1.7 | 1.2 |  |
| 0.3 | 1.1 | 1.4 | 1.1 | 1.1 | 1.7 | 1.2 | 1.7 | 1.7 | 0.9 | 1.2 | 1.9 | 1.4 |  |
| 0.1 | 1.1 | 1.8 | 1.1 | 1.2 | 1.6 |  | 1.7 | 1.5 | 0.9 | 1.2 | 1.9 | 1.4 |  |
| -0.05 | 1.1 | 1.6 | 1.0 | 1.2 | 1.7 |  | 1.7 | 1.7 | 1.0 | 1.2 | 1.9 | 1.3 |  |

**Table S6.** The exact S2p_3/2_ and P2p_3/2_ binding energy positions. The applied shift correction is based on BE_V_ – ΔBE = BE_OCP_, representing the exact BE position of the fitted compounds without voltage-dependent shift. As reduced compounds were not present at OCP, their shift has been corrected respectively to the ΔBE shift of the S-P compound.

| **Binding energy position (eV) at OCP: BE_OCP_ = BE_V_ – ΔBE** | | | | | | | | |
| --- | --- | --- | --- | --- | --- | --- | --- | --- |
| S2p 3/2 | | | P2p 3/2 | | | | | |
| S-P | P-S-S-P | Li_2_S | S-P | P-S-S-P | Li_x_P | Li_y_P | Li_z_P | Li_3_P |
| 161.5 | 162.9 | 160.0 | 131.7 | 132.6 | 130.2 | 128.1 | 126.7 | 124.6 |


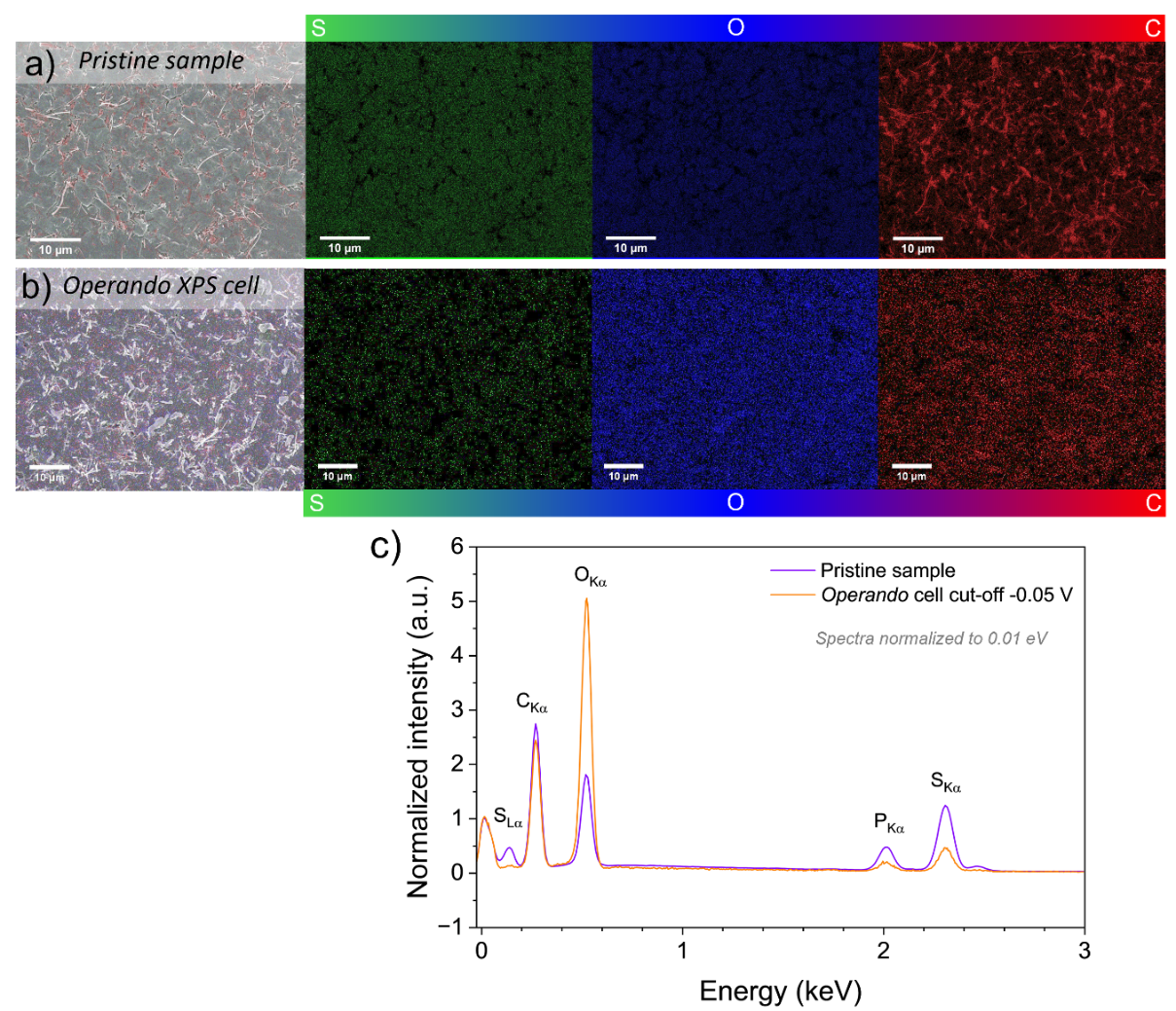


**Figure S9.** In-Lens SEM images and their corresponding EDX maps acquired on (LPS:VGCF, 80:20 wt%) WE, with sulfur (green), oxygen (blue) and carbon (red) for (a) the pristine WE pellet and (b) the WE pellet after reduction to -0.05 V inside the operando XPS cell. (c) The corresponding energy dispersive sum spectra for the EDX maps above in (a) and (b) are shown. All spectra have been normalized to the intensity at 0.01 eV.


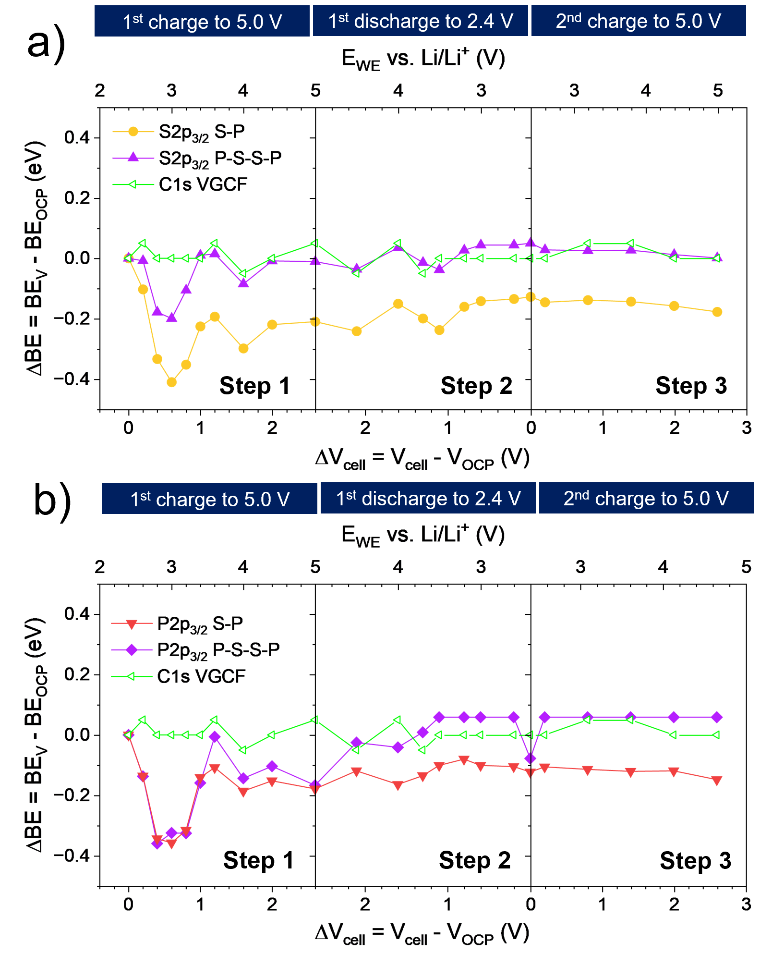


**Figure S10.** The changes in the binding energy (BE) position ΔBE dependent on the difference of applied cell voltage (ΔV_cell_) are shown for the high voltage range for steps 1, 2, and 3. In (a) the S2p_3/2_ BE shift for S-P (yellow), P-S-S-P (purple), and C1s of VGCF (green) are shown. In (b) the P2p_3/2_ BE shift for S-P (red), P-S-S-P (purple), and C1s of VGCF (green) are shown.


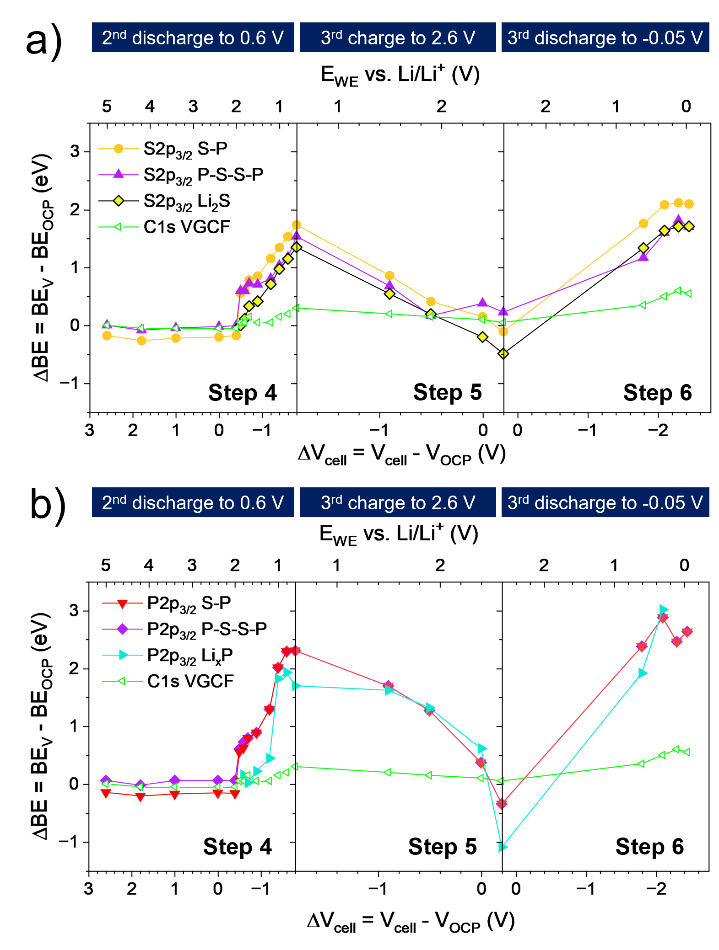


**Figure S11.** The changes in the binding energy (BE) position ΔBE dependent on the difference of applied cell voltage (ΔV_cell_) are shown for the low voltage range for steps 4, 5, and 6. In (a) the S2p_3/2_ BE shift for S-P (yellow), P-S-S-P (purple), Li_2_S (black-yellow), and C1s of VGCF (green) are shown. In (b) the P2p_3/2_ BE shift for S-P (red), P-S-S-P (purple), Li_x_P (light blue), and C1s of VGCF (green) are shown.

**Table S7.** Summary of ionic and electrical conductivities for commonly observed degradation byproducts of sulfide SEs.

| **Compound** | **Ionic conductivity**  **(S cm^-1^)** | **Electrical conductivity**  **(S cm^-1^)** | **Reference** |
| --- | --- | --- | --- |
| Li_2_O | 10^-9^ – 10^-12^ | 10^-14^ | ^[11]^ |
| Li_2_S | 10^-9^ – 10^-13^ | 10^-8^ – 10^-9^ | ^[12]^, ^[13]^ |
| Li_3_P (crystalline) | 10^-4^ – 10^-5^ | 10^-4^ | ^[14]^ |
| S^0^ | -- | 10^-18^ – 10^-30^ | ^[13, 15]^ |
| P^0^ (red) | -- | 10^-12^ | ^[16]^ |
| P_2_S_5_ | -- | 10^-10^ | Measured in this work |
| Li_3_PS_4_ | 10^-4^ | 10^-9^ | ^[17]^/Measured in this work |
| Li_2_P_2_S_6_ (reductive species) | 10^-11^ | Reported as low | ^[9, 18]^ |

*Calculation of theoretical volume changes*

We collected the volume of the reported species from *Wang et al.*^[5]^ As we did not detect elemental S^0^ we assume the compound Li_4_P_2_S_8_ to be present as P-S-S-P at 5.0 V, for which we assume a density of 2.0 g cm^-3^ based on common values for bridging sulfur species reported by *Lee et al.*^[19]^

2 Li_3_PS_4_ 🡪 Li_4_P_2_S_8_ + 2 Li^+^ + 2 e^-^ (**Equation S2a**)

Vol(LPS) = 94.8 cm^3^ per mol; Vol(Li_4_P_2_S_8_) = 173.1 cm^3^ per mol (**Equation S2b**)

ΔVol = -8.7 % (**Equation S2c**)

When reducing from 5.0 V to 0.6 V, the following reaction is assumed based on our observation in *operando* XPS and assuming full reduction of phosphorus to Li_3_P:

Li_4_P_2_S_8_ + 2 Li^+^ + 2 e^-^ 🡪 2 Li_3_PS_4_ (**Equation S3a**)

2 Li_3_PS_4_ + 16 Li^+^ + 16 e^-^ 🡪 8 Li_2_S + 2 Li_3_P (**Equation S3b**)

Vol(Li_4_P_2_S_8_) = 173.1 cm^3^ per mol; Vol(LPS) = 94.8 cm^3^ per mol; Vol(Li_2_S) = 27.7 cm^3^ per mol; Vol(Li_3_P) = 36.2 cm^3^ per mol (**Equation S3c**)

ΔVol = +70 % (**Equation S3d**)

An increase in the volume is in line with our experimentally observed data in Figure 7, however, its relative magnitude compared to oxidation appears much more than what we observe at 1.0 V cut-off potential. Hence, we made the additional effort to calculate the volume changes based on the byproducts' atomic percent (at%) fraction extracted from *operando* XPS in **Table S2**. Based on the at% at 5.0 V we balance our oxidation equation as follows:

17 Li_3_PS_4_ 🡪 3 Li_3_PS_4_ + 7 Li_4_P_2_S_8_ + 20 Li^+^ + 20 e^-^ (**Equation S4**)

with a volume for 17 mol Li_3_PS_4_ = 1611.6 cm^3^; and a volume for 3 mol LPS and for 7 mol Li_4_P_2_S_8_ = 1496.2 cm^3^. The total change in volume upon oxidation is around -7.2 %, which we can refer to as the -200 kPa in Figure 7a.

We repeated the same exercise for reduction from 5.0 V to 1.0 V (as the limit is set by the LTO anode on reduction), again balancing the reduction reaction with the at% ratio extracted from **Table S2** at 1.0 V, which is only partially possible, as the composition of bridging sulfur species might change upon discharge, giving an extra of 3 sulfur atoms, which cannot be accommodated in the current equation. Also, the compound assigned to P^2-^ oxidation state was not included, as there is no structural information available.

4/3 Li_3_PS_4_ + 7/3 Li_4_P_2_S_8_ + 22 Li^+^ + 22 e^-^ 🡪 2 Li_3_PS_4_ + Li_4_P_2_S_8_ + 5 Li_2_S + P^0^ + 0.5 LiP + 0.5 Li_3_P (+ x Li_y_S_z_) (**Equation S5**)

After balancing the reaction equation, the volume change upon reduction amounts to +1.9 %, which is closer to the detected +100 kPa pressure increase in Figure 7b. This concludes that our detected composition shows a stronger correlation with the measured pressure changes, than the simplified reaction mechanism summarized in **Equation S3**.


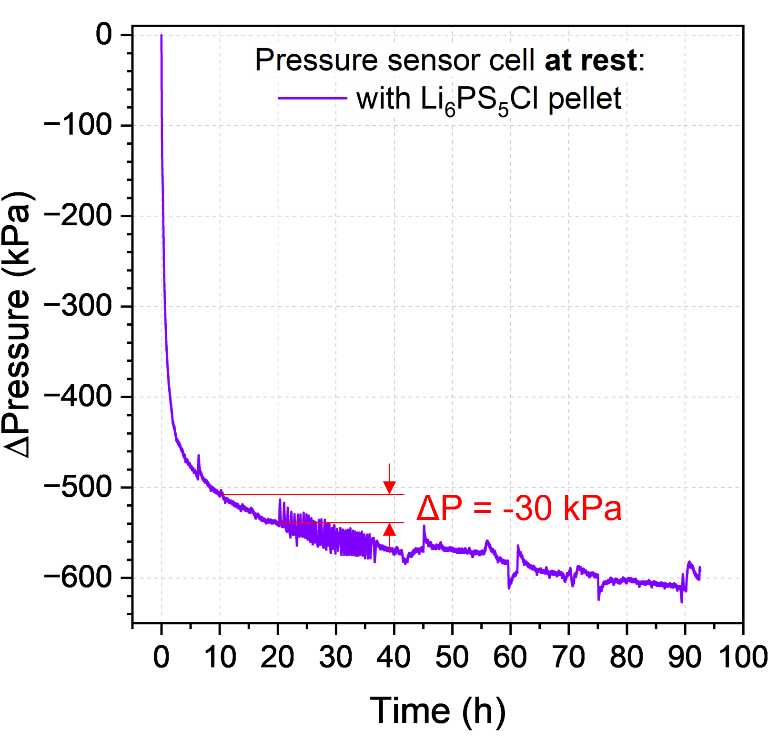


**Figure S12.** Pressure drop evolution over time inside the CompreFrame cell at rest caused by the cell mechanical relaxation, with Li_6_PS_5_Cl pellet inside. The pellet was pressed at 380 MPa, and the initial stack pressure was 80 MPa. Within 10 hours the relaxation is around -30 kPa.

**
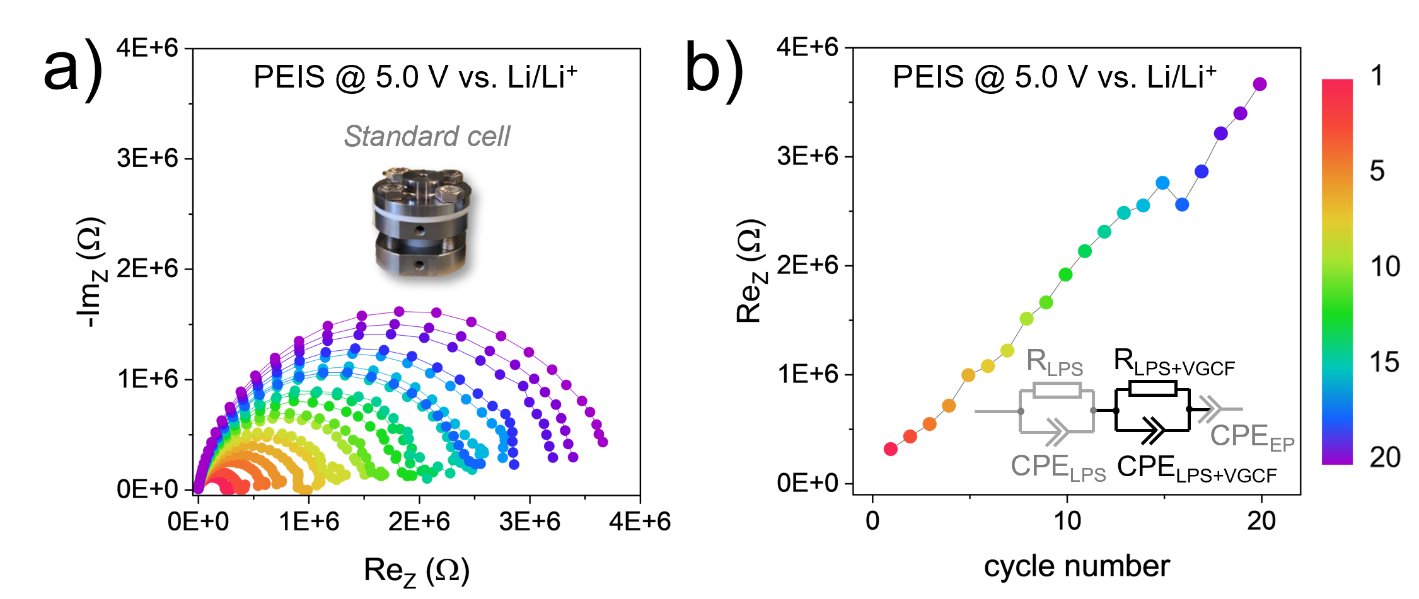
**

**Figure S13.** Standard electrochemical cell impedance evolution in the high voltage range. (a) Nyquist plot of 20 cycles with potentiostatic EIS at 5.0 V cutoff potential and the corresponding fitting of the interface resistance of the LPS+VGCF composite over the cycle number in (b) with the adequate equivalent circuit as inset (black highlighted R+CPE element indicating the LPS+VGCF composite contribution).


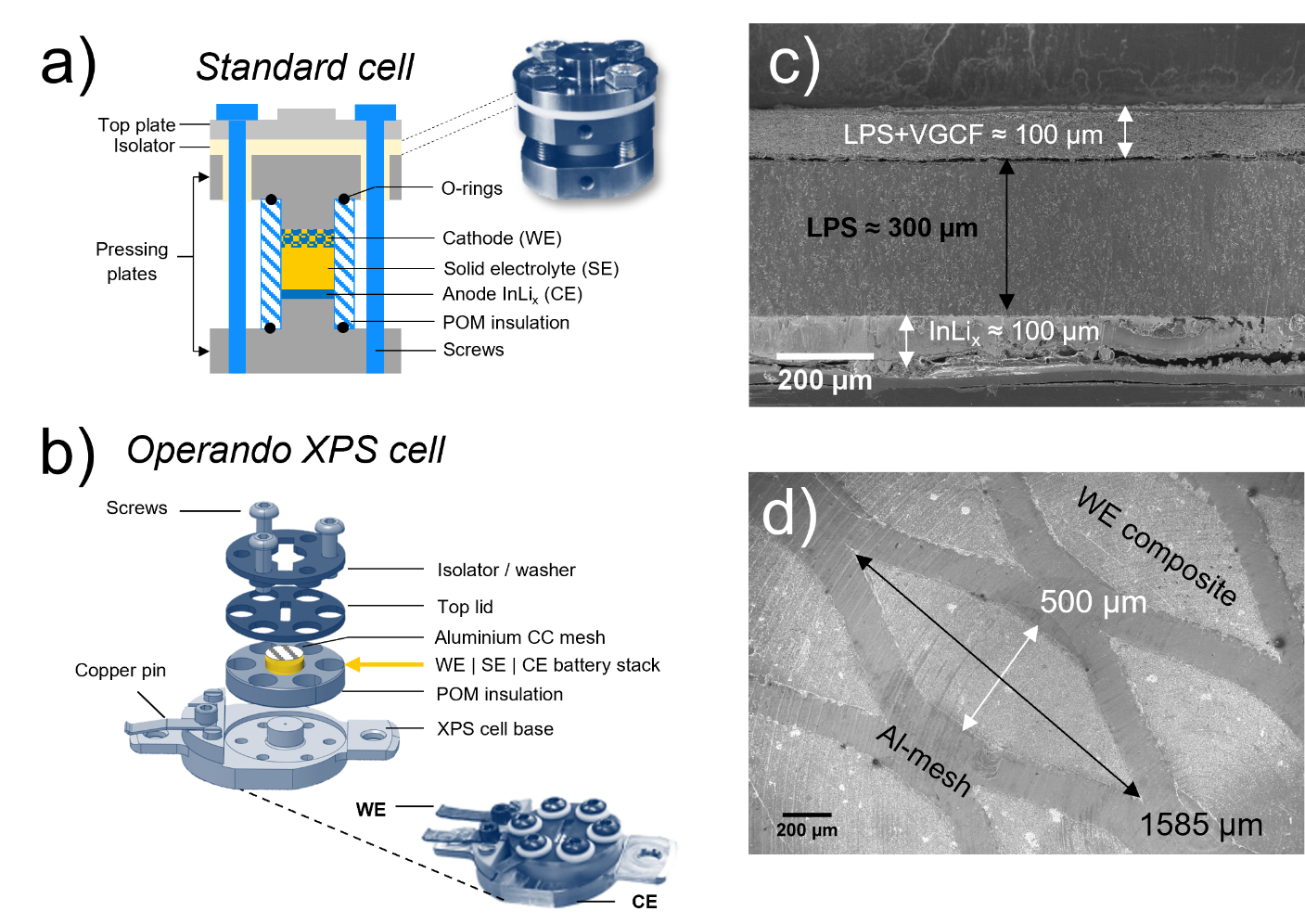


**Figure S14.** Standard electrochemical cell design in (a) and operando XPS cell design in (b). SEM cross-section after Ar-ion milling in (c) depicting the thickness of the full standard cell battery stack. In-plane In-lens SEM image of the operando XPS cell surface, including the WE composite with the current-collector Al-mesh in (d).


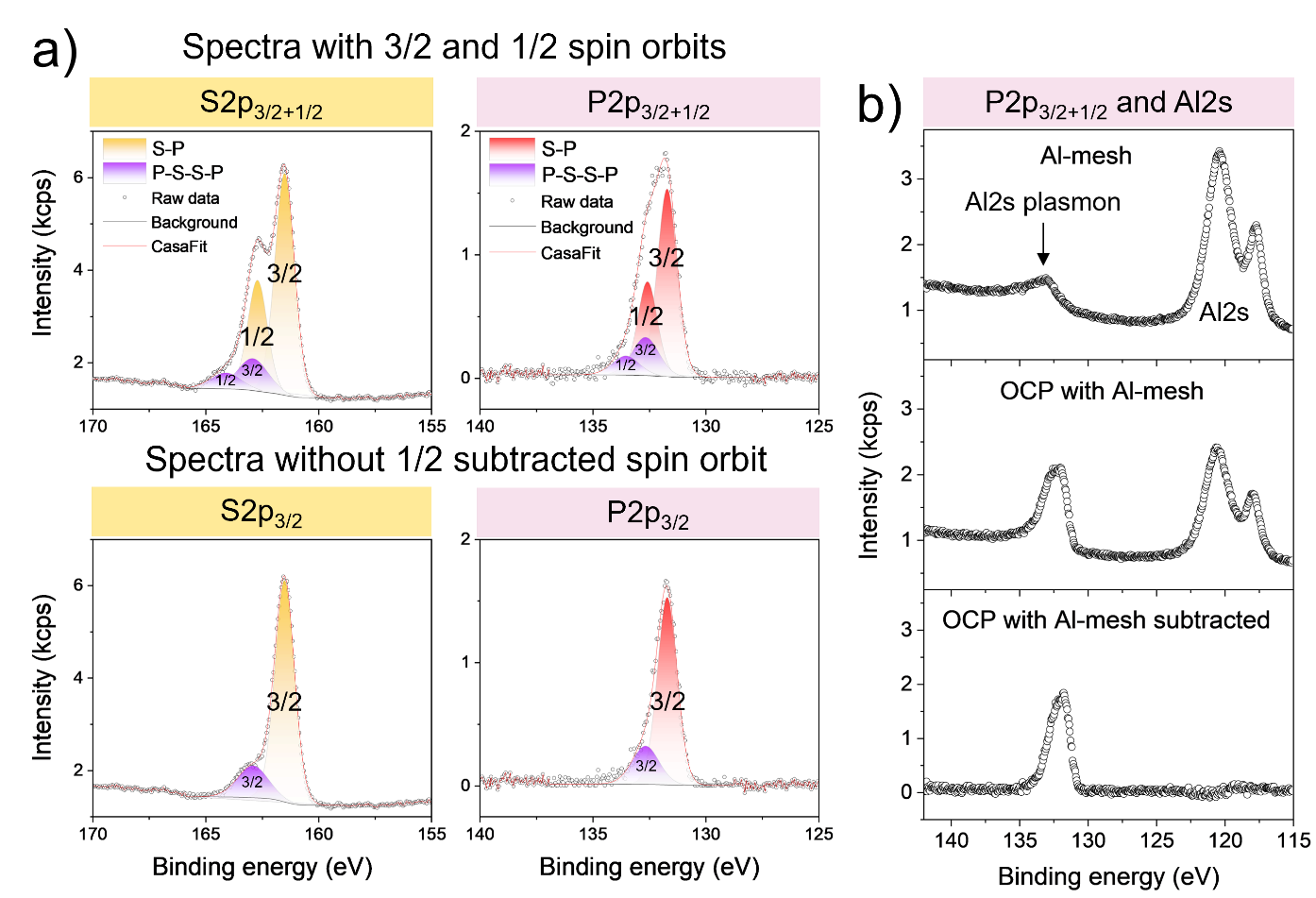


**Figure S15**. In (a) S2p and P2p spectra with 3/2 and 1/2 spin orbits for all compounds of the non-cycled WE composite, and their peak-deconvolution carried out in CasaXPS software are shown, with their corresponding 1/2 doublet-subtracted S2p_3/2_ and P2p_3/2_ peaks shown below. In (b) the background subtraction of the Al-mesh in the P2p spectra is shown for the composite WE (LPS:VGCF, 80:20 wt%) at OCP.

References

[1] A. Jablonski, C. Powell, *National Institute of Standards and Technology, Gaithersburg* **2010**. <https://doi.org/10.18434/T48C78>.

[2] J. Auvergniot, A. Cassel, J.-B. Ledeuil, V. Viallet, V. Seznec, R. Dedryvere, *Chemistry of Materials* **2017**, *29*, 3883-3890. <https://doi.org/10.1021/acs.chemmater.6b04990>.

[3] G. F. Dewald, S. Ohno, M. A. Kraft, R. Koerver, P. Till, N. M. Vargas-Barbosa, J. Janek, W. G. Zeier, *Chemistry of Materials* **2019**, *31*, 8328-8337. <https://doi.org/10.1021/acs.chemmater.9b01550>.

[4] D. H. S. Tan, E. A. Wu, H. Nguyen, Z. Chen, M. A. T. Marple, J.-M. Doux, X. Wang, H. Yang, A. Banerjee, Y. S. Meng, *ACS Energy Letters* **2019**, *4*, 2418-2427. <https://doi.org/10.1021/acsenergylett.9b01693>.

[5] S. Wang, M. Tang, Q. Zhang, B. Li, S. Ohno, F. Walther, R. Pan, X. Xu, C. Xin, W. Zhang, L. Li, Y. Shen, F. H. Richter, J. Janek, C. W. Nan, *Advanced Energy Materials* **2021**, *11*, 2101370. <https://doi.org/10.1002/aenm.202101370>.

[6] N. Erika, T. S. Arthur, P. Bonnick, K. Suto, M. John, *MRS Advances* **2019**, *4*, 2627-2634. <https://doi.org/10.1557/adv.2019.255>.

[7] S. Wang, W. Zhang, X. Chen, D. Das, R. Ruess, A. Gautam, F. Walther, S. Ohno, R. Koerver, Q. Zhang, W. G. Zeier, F. H. Richter, C. W. Nan, J. Janek, *Advanced Energy Materials* **2021**, *11*, 2100654. <https://doi.org/10.1002/aenm.202100654>.

[8] F. Walther, S. Randau, Y. Schneider, J. Sann, M. Rohnke, F. H. Richter, W. G. Zeier, J. Janek, *Chemistry of Materials* **2020**, *32*, 6123-6136. <https://doi.org/10.1021/acs.chemmater.0c01825>.

[9] R. Koerver, F. Walther, I. Aygün, J. Sann, C. Dietrich, W. G. Zeier, J. Janek, *Journal of Materials Chemistry A* **2017**, *5*, 22750-22760. <https://doi.org/10.1039/c7ta07641j>.

[10] H. Q. Nguyen, M. D. Kanedal, J. Todt, F. Jin, Q. Do, D. Zalka, A. Maximenko, D. Stoian, N. Schell, W. van Beek, *Journal of the American Chemical Society* **2024**, *147*, 23492–23503.

[11] S. Lorger, R. Usiskin, J. Maier, *Journal of The Electrochemical Society* **2019**, *166*, A2215-A2220. <https://doi.org/10.1149/2.1121910jes>.

[12] S. Choi, I. Yoon, W. T. Nichols, D. Shin, *Ceramics International* **2018**, *44*, 7450-7453. <https://doi.org/10.1016/j.ceramint.2018.01.104>.

[13] F. Zhang, Y. Luo, X. Gao, R. Wang, *ACS Sustainable Chemistry & Engineering* **2020**, *8*, 12100-12109. <https://doi.org/10.1021/acssuschemeng.0c03425>.

[14] J. Li, D. Liu, H. Sun, D. Qu, Z. Xie, H. Tang, J. Liu, *SmartMat* **2023**, *4*. <https://doi.org/10.1002/smm2.1200>.

[15] P. T. Dirlam, R. S. Glass, K. Char, J. Pyun, *Journal of Polymer Science Part A: Polymer Chemistry* **2017**, *55*, 1635-1668. <https://doi.org/10.1002/pola.28551>.

[16] Z. Cheng, Y. Wu, H. Huang, *Solid State Ionics* **2023**, *389*, 116098. <https://doi.org/10.1016/j.ssi.2022.116098>.

[17] T. P. Poudel, M. J. Deck, P. Wang, Y. Y. Hu, *Advanced Functional Materials* **2024**, *34*, 2309656. <https://doi.org/10.1002/adfm.202309656>.

[18] C. Dietrich, D. A. Weber, S. Culver, A. Senyshyn, S. J. Sedlmaier, S. Indris, J. r. Janek, W. G. Zeier, *Inorganic chemistry* **2017**, *56*, 6681-6687. <https://doi.org/10.1021/acs.inorgchem.7b00751>.

[19] B. Lee, K. Jun, B. Ouyang, G. Ceder, *Chemistry of Materials* **2023**, *35*, 891-899. <https://doi.org/10.1021/acs.chemmater.2c02458>.
